# Resistant starch can improve insulin sensitivity independently of the gut microbiota

**DOI:** 10.1186/s40168-017-0230-5

**Published:** 2017-02-07

**Authors:** Laure B. Bindels, Rafael R. Segura Munoz, João Carlos Gomes-Neto, Valentin Mutemberezi, Inés Martínez, Nuria Salazar, Elizabeth A. Cody, Maria I. Quintero-Villegas, Hatem Kittana, Clara G de los Reyes-Gavilán, Robert J. Schmaltz, Giulio G. Muccioli, Jens Walter, Amanda E. Ramer-Tait

**Affiliations:** 1 Department of Food Science and Technology, University of Nebraska-Lincoln, Lincoln, NE USA; 2 Bioanalysis and Pharmacology of Bioactive Lipids Research Group, Louvain Drug Research Institute, Université catholique de Louvain, Brussels, Belgium; 3 Department of Agricultural, Food and Nutritional Science, University of Alberta, Edmonton, Alberta Canada; 4 Department of Microbiology and Biochemistry of Dairy Products, Instituto de Productos Lácteos de Asturias-Consejo Superior de Investigaciones Científicas (IPLA-CSIC), Asturias, Spain; 5 Department of Biological Sciences, University of Alberta, Edmonton, Alberta Canada

**Keywords:** Resistant starch, Gut microbiota, Insulin sensitivity, Adipose tissue macrophages

## Abstract

**Background:**

Obesity-related diseases, including type 2 diabetes and cardiovascular disease, have reached epidemic proportions in industrialized nations, and dietary interventions for their prevention are therefore important. Resistant starches (RS) improve insulin sensitivity in clinical trials, but the mechanisms underlying this health benefit remain poorly understood. Because RS fermentation by the gut microbiota results in the formation of physiologically active metabolites, we chose to specifically determine the role of the gut microbiota in mediating the metabolic benefits of RS. To achieve this goal, we determined the effects of RS when added to a Western diet on host metabolism in mice with and without a microbiota.

**Results:**

RS feeding of conventionalized mice improved insulin sensitivity and redressed some of the Western diet-induced changes in microbiome composition. However, parallel experiments in germ-free littermates revealed that RS-mediated improvements in insulin levels also occurred in the absence of a microbiota. RS reduced gene expression of adipose tissue macrophage markers and altered cecal concentrations of several bile acids in both germ-free and conventionalized mice; these effects were strongly correlated with the metabolic benefits, providing a potential microbiota-independent mechanism to explain the physiological effects of RS.

**Conclusions:**

This study demonstrated that some metabolic benefits exerted by dietary RS, especially improvements in insulin levels, occur independently of the microbiota and could involve alterations in the bile acid cycle and adipose tissue immune modulation. This work also sets a precedent for future mechanistic studies aimed at establishing the causative role of the gut microbiota in mediating the benefits of bioactive compounds and functional foods.

**Electronic supplementary material:**

The online version of this article (doi:10.1186/s40168-017-0230-5) contains supplementary material, which is available to authorized users.

## Background

Obesity has become a public health crisis, with an estimated 39% of adults ages 18 and over worldwide considered overweight in 2014 and 13% deemed obese [1]. Many obese individuals also experience a cluster of abnormalities (elevated blood triglycerides, fasting hyperglycemia, high blood pressure, and reduced HDL cholesterol), referred to as the metabolic syndrome, that places them at significant risk for the development of type 2 diabetes (T2D), hypertension, dyslipidemia, and cardiovascular disease (CVD) [2]. Resistance to insulin is considered the earliest detectable metabolic aberrancy in persons who eventually develop T2D [3]. The mechanisms underlying insulin resistance are multifactorial and include ectopic lipid accumulation in the muscle and liver as well as systemic inflammation, especially in adipose tissue [3]. In particular, adipose tissue macrophages (ATMs) have been identified as immune cells driving the development of insulin resistance [4]. Considering these underlying factors, successful therapeutic approaches and dietary interventions for treating metabolic syndrome should ideally provide both metabolic and immunological benefits to the host.

Dietary fibers, such as resistant starches (RS), have been studied extensively and are promising nutritional interventions for preventing metabolic diseases [5–7]. RS in particular are well documented to modulate insulin sensitivity in healthy and obese volunteers as well as in patients with metabolic syndrome [8–11]. Although these benefits likely arise from a multitude of mechanisms, the gut microbiota is increasingly considered one of the key factors underlying these health benefits [12–15]. Recent research concerning the beneficial effects of short-chain fatty acids (SCFA), which result from bacterial fermentation of non-digestible carbohydrates such as RS, on intestinal barrier function, gut peptide secretion [16–18], and immune function [19–21] further strengthen the concept that dietary fibers may exert some of their metabolic benefits via gut bacteria. However, despite the rationale for considering the microbiota in mediating the physiological effects of RS and fiber, very few studies have taken a mechanistic approach to determine the exact role of the microbiota such as feeding germ-free mice or performing microbiota transplants [22–25]. Rather, most studies have only assessed bacterial participation in a phenotype by correlations and not by methods that establish causality [26–30].

To systematically determine the gut microbiota’s contribution to the health benefits of dietary fiber, we compared the effects of feeding RS on host metabolism in Western diet-fed mice with and without a microbiota. The two types of RS preparations (RS type 2 and RS type 4) selected for this study have both been shown to significantly impact host metabolism [9, 11, 31, 32], yet each elicits very distinct effects on human gut microbiota composition [33]. We therefore sought to evaluate how two types of RS capable of differentially influencing the gut microbiota impact insulin sensitivity. The findings reported here demonstrate that some metabolic benefits mediated by dietary RS, especially improvements in insulin levels, occur independently of the gut microbiota and could potentially involve alterations in the bile acid cycle as well as adipose tissue immune modulation in the form of macrophage recruitment and retention.

## Results

### Germ-free C3H mice experienced similar gains in body weight as their conventionalized counterparts when fed a Western diet

**Fig. 1:**
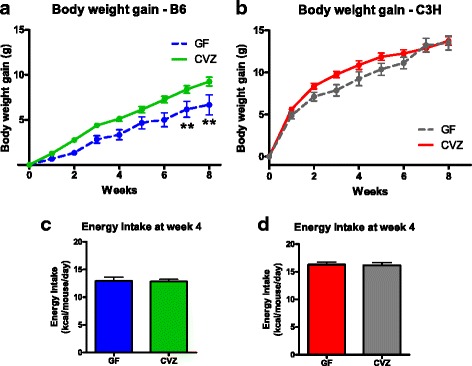
Germ-free C3H mice experienced similar gains in body weight as their conventionalized counterparts when fed a Western diet. Body weight gain in germ-free (*GF*) and conventionalized (*CVZ*) male C57BL/6 (*B6*) mice (**a**) and GF and CVZ male C3H/HeN (*C3H*) mice (**b**) fed a WD. Energy intake measured at week 4 in GF and CVZ B6 mice (**c**) and GF and CVZ C3H mice (**d**) fed a WD. **p < 0.01 versus CVZ (two-way ANOVA with repeated measures and Bonferroni post hoc tests). Mean ± SEM. **a** *N* = 6–8 mice. **b** *N* = 8 mice. **c** *N* = 4 cages. **d** *N* = 3 cages

To directly test the role of the gut microbiota in mediating the metabolic benefits of RS, we sought to compare the effects of feeding RS to mice with and without a gastrointestinal microbiota. However, C57BL/6 (B6) mice, the mouse strain traditionally used for diet-induced obesity (DIO) research, are resistant to the effects of feeding a Western diet (WD) when maintained germ-free [34–36]. In contrast, germ-free (GF) C3H/HeN (C3H) mice are generally not protected against DIO [37]. To compare the role of the gut microbiota in the development of DIO in the two strains of mice, GF and conventionalized (CVZ) C3H and B6 mice were all fed the same WD for 8 weeks. GF B6 mice gained significantly less weight compared to their conventionalized counterparts (Fig. 1a). In contrast, GF C3H mice gained weight similarly to CVZ controls (Fig. 1b). Of note, CVZ C3H mice gained more weight and exhibited a higher energy intake than CVZ B6 mice (Fig. 1). These findings are consistent with previous reports [34–37] and indicate that GF C3H mice are a tractable model in which to study the role of the microbiota in the therapeutic effects of a dietary fiber in the context of obesity.

**Fig. 2:**
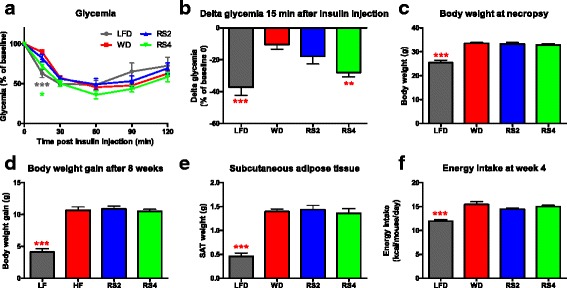
Resistant starches improved insulin sensitivity in C3H mice in the presence of a microbiota. Glycemic response after insulin injection in conventionalized 15-week-old C3H/HeN (C3H) mice fed experimental diets for 7 weeks (**a**, **b**) (*LFD* low-fat diet, *WD* Western diet, *RS2* WD with resistant starch 2, *RS4* WD with resistant starch 4). Body weights at necropsy (**c**), body weight gain over 8 weeks of dietary intervention (**d**), subcutaneous adipose tissue (SAT) weights at necropsy (**e**), and energy intake (as recorded over a week at week 4) (**f**) in conventionalized C3H mice fed experimental diets for 8 weeks. *p < 0.05, **p < 0.01, ***p < 0.001 versus WD (one-way ANOVA with Dunnett’s post hoc tests). Mean ± SEM. **a**, **b** *N* = 6–9. **c**–**e** *N* = 8–9. **f** *N* = 4 cages

Because C3H mice fed a Western diet gained weight independently of the gut microbiota, we next sought to determine whether C3H mice respond to RS supplementation similarly to B6 mice (in which weight gain was gut microbiota-dependent) in the presence of a microbiota. C3H and B6 mice were fed either a control WD or a WD enriched with 10% RS for 8 weeks (Table 1). A portion of the corn starch in the purified diets was replaced with RS—an approach often applied in human nutritional intervention trials. In those interventions, participants are asked to consume either RS-enriched products [33, 38, 39] or RS supplements [9–11, 40, 41] with native or readily digestible starch or starch-based products serving as the placebo. The two types of RS fed (resistant starch type 2 (RS2) and resistant starch type 2 (RS4)) in this study were selected because they are known to improve host metabolism (including insulin resistance) yet elicit distinct effects on human gut microbiota composition [9, 11, 31–33]. In C3H mice, supplementation with RS4 improved insulin sensitivity despite having no impact on body weight, body weight gain, subcutaneous adipose tissue weight, and energy intake (Fig. 2). Similar results were obtained in B6 mice (Additional file 1: Fig. S1). These experiments revealed that, although the influence of the microbiota on weight gain was line-dependent, animals from both lines maintained the ability to respond to dietary modulation by RS in the presence of a microbiota.

**Table 1:** Composition of the experimental diets. HI-MAIZE® 260 and Fibersym® RW contain 62.5 and 89.1% RS, respectively. See Additional file 3: Table S4 for complete details

	LFD	WD	RS2	RS4
	g (%)	g (%)	g (%)	g (%)
Protein	19.2	23.7	23.7	23.7
Carbohydrate	67.3	46.1	46.1	46.1
Fat	4.3	23.6	23.6	23.6
	kcal (%)	kcal (%)	kcal (%)	kcal (%)
Protein	20	20	21.9	21.9
Carbohydrate	70	34.1	28	28
Fat	10	44.9	49.1	49.1
Ingredient quantity	g	g	g	g
Casein, 30 mesh	200	200	200	200
l-cystine	3	3	3	3
Corn starch	550	137.3	0	41
Maltodextrine 10	150	35.5	35.5	35.5
Sucrose	0	172.8	172.8	172.8
HiMaize (RS2)	0	0	137.3	0
Fibersym (RS4)	0	0	0	96.3
Cellulose BW200	50	50	50	50
Soybean oil	25	25	25	25
Lard	20	177.5	177.5	177.5
Mineral mix S10026	10	10	10	10
Dicalcium phosphate	13	13	13	13
Calcium carbonate	5.5	5.5	5.5	5.5
Potassium citrate, 1H_2_O	16.5	16.5	16.5	16.5
Vitamin mix V1001	10	10	10	10
Choline bitartrate	2	2	2	2
Dyes	0.05	0.05	0.05	0.05
Energy density (kcal/g)	3.85	4.73	4.33	4.33

### Feeding resistant starches substantially altered the gut microbiota composition

**Fig. 3:**
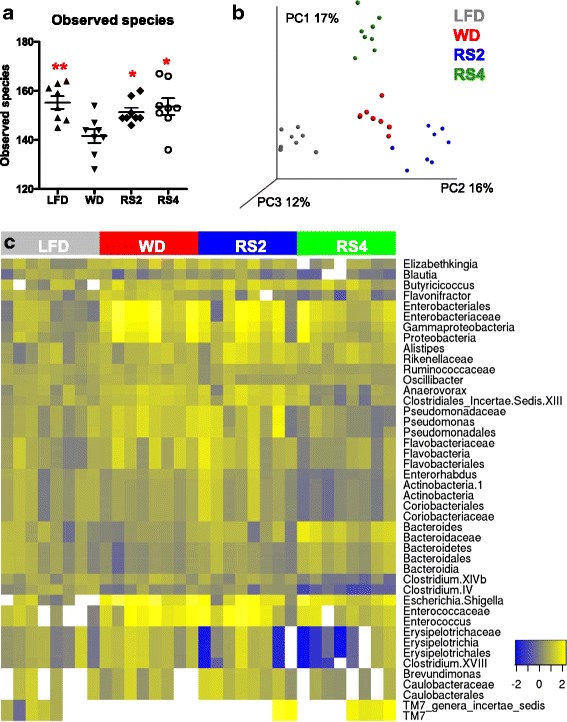
Resistant starches changed the gut microbiota composition in conventionalized C3H mice fed experimental diets for 8 weeks. **a** Alpha-diversity index. **b** Principal Coordinate Analysis plot of β-diversity based on binary Jaccard distance. **c** Log-fold change in the relative abundance of taxa significantly affected by the dietary intervention (adjusted *p* value <0.05). *LFD* low-fat diet, *WD* Western diet, *RS2* WD with resistant starch 2, *RS4* WD with resistant starch 4. *p < 0.05, **p < 0.01 versus WD (one-way ANOVA with Dunnett’s post hoc tests). Mean ± SEM. *N* = 8

Supplementing a WD with RS2 or RS4 restored the microbial α-diversity reduced by feeding a WD to CVZ C3H mice (Fig. 3a). Principal Coordinate Analysis (PCoA) plot of β-diversity based on binary Jaccard distance revealed that gut communities clustered according to diet (Fig. 3b). Similar results were obtained via two other independent β-diversity metrics (Bray-Curtis distance and unweighted UniFrac distance, Additional file 2: Fig. S2a, b). Together, these findings indicate that RS induced global changes to the structure of the gut microbiota in mice. Accordingly, parametric analyses revealed that 124 species-like OTUs and 46 taxa were significantly affected by dietary treatment (Fig. 3c; Additional file 3: Tables S1, S2). Interestingly, RS corrected the proportions of seven taxa (Ruminococcaceae and Clostridiales Incertae Sedis XIII families and *Clostridium* IV, *Anaerovorax*, *Oscillibacter*, *Elizabethkingia*, and *Flavonifractor* genera) whose levels were altered upon feeding a WD. Consistent with observations from a previous RS feeding study of healthy humans, RS4 increased the Bacteroidetes phylum and decreased the Ruminococcaceae and Erysipelotrichaceae families [33]. Non-parametric analysis of bacterial taxa using LEfSe confirmed most of these results and also highlighted that Proteobacteria-related taxa were specifically enriched in the WD-fed mice (Additional file 2: Fig. S2c, d). Altogether, these data indicate that supplementing a WD with RS caused substantial shifts in gut microbiota composition and was able to redress some of the aberrancies induced by feeding a WD.

### Feeding resistant starches improved plasma insulin levels and the index of insulin resistance independently of the gut microbiota in C3H mice

To directly test the role of the gut microbiota in mediating the metabolic benefits of RS, both GF and CVZ C3H mice were fed either a control WD or a WD enriched with 10% RS for 8 weeks (Table 1). We choose to perform this experiment with C3H mice because, unlike the B6, GF C3H mice gained weight similarly to their CVZ littermates when fed a WD. Although WD feeding increased body weight, white adipose tissue weight, and plasma leptin levels in both GF and CVZ mice compared to low-fat diet (LFD) fed controls, RS feeding did not significantly impact these parameters (Additional file 4: Fig. S3a–c). No changes were observed in lean mass (as measured by tibialis muscle weights) for mice on any treatment (Additional file 4: Fig. S3d).

**Fig. 4:**
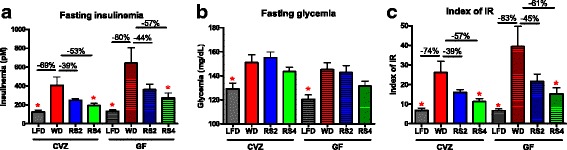
Resistant starches improved plasma insulin levels and the index of insulin resistance in conventionalized (CVZ) and germ-free (GF) C3H mice fed experimental diets for 8 weeks. **a** Plasma insulin levels in 6-h fasted mice. **b** Plasma glucose levels in 6-h fasted mice. **c** Index of insulin resistance (*IR*), also known as HOMA-IR. *LFD* low-fat diet, *WD* Western diet, *RS2* WD with resistant starch 2, *RS4* WD with resistant starch 4. *p < 0.05 versus WD fed mice of the same microbial status (one-way ANOVA with Dunnett’s post hoc tests). Microbial status significantly impacted fasting insulinemia and glycemia (analysis of all eight treatments using two-way ANOVA). Mean ± SEM. *N* = 7–8

**Fig. 5:**
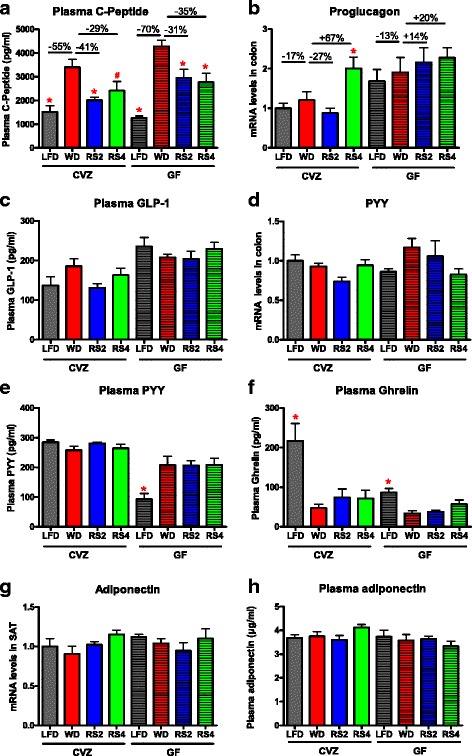
Gut peptide and hormone levels in germ-free (*GF*) and conventionalized (*CVZ*) C3H mice fed experimental diets for 8 weeks. **a** Fasting plasma C-peptides levels. **b** Proglucagon mRNA expression in the colon. **c** Fasting plasma glucagon-like peptide 1 (GLP-1) levels. **d** PYY mRNA expression in the colon. **e** Fasting plasma peptide YY (PYY) levels. **f** Fasting plasma ghrelin levels. **g** Adiponectin mRNA expression in the subcutaneous adipose tissue (SAT). **h** Fasting plasma adiponectin levels. Mice were fasted for 6 h prior to sampling. *LFD* low-fat diet, *WD* Western diet, *RS2* WD with resistant starch 2, *RS4* WD with resistant starch 4. ^#^ *p* = 0.07, *p < 0.05 versus WD fed mice of the same microbial status (one-way ANOVA with Dunnett’s post tests). Microbial status significantly impacted C-peptide, GLP-1, PYY, and ghrelin levels, as well as proglucagon mRNA expression (analysis of all eight treatments using two-way ANOVA). Mean ± SEM. *N* = 7–8, except for **c** and **f** where *N* = 6–8

Both GF and CVZ C3H mice fed a control WD experienced a significant increase in fasting plasma insulin and glucose levels compared to the LFD-fed control animals (Fig 4a, b). Feeding a RS4-supplemented WD significantly decreased plasma insulin levels in CVZ mice (Fig. 4a). Moreover, these improvements were associated with substantial shifts in gut microbiota composition in CVZ mice (described above). Such associations are often considered an indication that the gut microbiota mediates the health effects of a dietary intervention [27, 42, 43]. However, when a WD supplemented with RS4 was fed to GF C3H mice, plasma insulin levels were significantly decreased to levels consistent with those observed in their CVZ counterparts, demonstrating that this RS-mediated metabolic improvement was not dependent on the presence of a gut microbiota (Fig. 4a). Both GF and CVZ mice fed an RS2-supplemented WD also experienced a similar trend toward decreased insulin levels, although it did not reach statistical significance (*p* = 0.07 for CVZ and *p* = 0.09 for GF, Dunnett’s post hoc tests). Hyperglycemia induced by the WD was not improved with RS feeding (Fig. 4b). Finally, feeding RS4 significantly improved the index of insulin resistance independently of the gut microbiota; feeding RS2 showed a similar trend (*p* = 0.07 for CVZ and *p* = 0.13 for GF, Dunnett’s post hoc tests) (Fig. 4c). Dietary supplementation with RS2 and RS4 also significantly improved circulating levels of C-peptide, a protein derived from proinsulin and a measure of insulin secretion, independently of microbial status (Fig. 5a).

Several reports indicate a role for the incretin glucagon-like peptide 1 (GLP-1) and peptide YY (PYY) in bringing about the metabolic benefits of RS, especially reductions in body weights and fat mass accumulation [32]. To that end, we measured the colonic messenger RNA (mRNA) expression and plasma levels of molecules related to the regulation of these incretins. Colonic expression of proglucagon, the precursor of GLP-1, was increased by RS4 feeding in a microbiota-dependent manner (Fig. 5b); however, no significant correlations (at the *q* value level) between proglucagon mRNA expression and any microbial taxa were found (data not shown). Proglucagon expression was increased in GF mice compared to CVZ littermates. In accordance with the increase in colonic proglucagon mRNA expression, active GLP-1 levels in plasma were also increased in GF mice (Fig. 5c) as previously described [44]. However, the RS4-induced increase in proglucagon mRNA expression was not accompanied by a change in active GLP-1 plasma levels (Fig. 5c). This apparent discrepancy between proglucagon mRNA expression and plasma GLP-1 levels has been reported in other studies and could be explained by several factors, such as differences in the activity of the dipeptidyl peptidase 4 or the prohormone convertase 1/3 [44, 45]. Colonic expression and plasma levels of PYY were not affected by the RS diets (Fig. 5d, e). Of note, PYY levels were globally lower in GF mice compared to CVZ counterparts, consistent with previous reports [46]. WD feeding decreased active ghrelin levels in both GF and CVZ animals compared to the LFD controls, but RS feeding had no additional impact (Fig. 5f). Neither plasma nor subcutaneous adipose tissue mRNA levels of adiponectin were affected by the dietary treatments, suggesting that adiponectin does not play a role in the modulation of insulin sensitivity by RS (Fig. 5g, h).

### Reduced expression of adipose tissue macrophage markers in RS-fed mice was associated with improvements in the index of insulin resistance

**Fig. 6:**
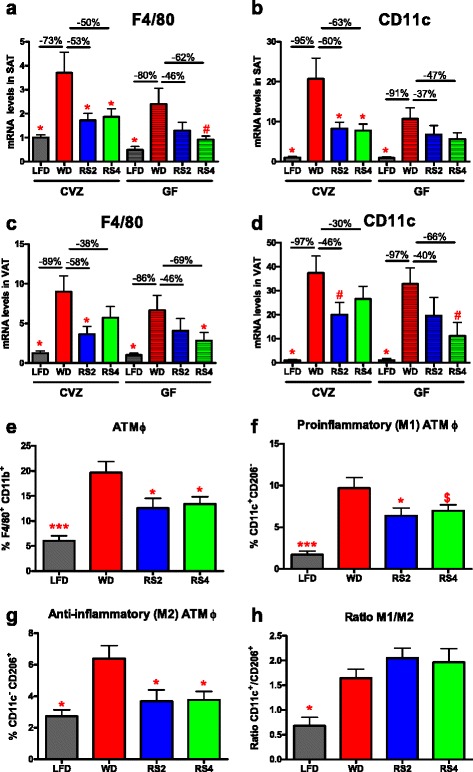
Altered expression of macrophage markers in the adipose tissues of C3H mice fed a Western diet supplemented with resistant starches for 8 weeks. **a**–**d** F4/80 and CD11c expression in the subcutaneous and visceral adipose tissues (SAT and VAT). **e**–**h** Flow cytometry analysis of the stromal vascular fraction (SVF) isolated from the SAT of conventionalized C3H mice. **e** Percentage of macrophages (F4/80^+^ CD11b^+^). **f** Percentage of M1 (CD11c^+^) macrophages. **g** Percentage of M2 (CD206^+^) macrophages. **h** Ratio of M1/M2, using CD11c as a M1 marker and CD206 as a M2 marker. *LFD* low-fat diet, *WD* Western diet, *RS2* WD with resistant starch 2, *RS4* WD with resistant starch 4. ^$^ *p* = 0.11, ^#^ *p* = 0.05–0.07, *p < 0.05, ***p < 0.001 versus WD fed mice of the same microbial status (one-way ANOVA with Dunnett’s post hoc tests). Analysis performed after normalization by log-transformation for **d** (GF data) and **f**. Microbial status significantly impacted F4/80 expression in SAT (analysis of all eight treatments using two-way ANOVA). Mean ± SEM. **a**–**d** *N* = 5–8. **e**–**h** *N* = 14–15 except for the LFD where *N* = 6

To begin dissecting how RS could modulate host physiological and metabolic characteristics independently of the microbiota, we examined markers of adipose tissue macrophages (ATM) as these innate immune cells and their polarization phenotypes have been implicated in the control of insulin sensitivity [4, 47]. Feeding RS4 significantly reduced subcutaneous adipose tissue (SAT) mRNA expression of F4/80, a tissue macrophage marker, in both GF and CVZ mice compared to WD-fed controls (Fig. 6a). SAT expression of CD11c, a marker of antigen presenting cells and M1 macrophages, was reduced by RS2 and RS4 feeding in CVZ mice; a similar change (albeit lower and not statistically significant) was also observed in GF mice (Fig. 6b). F4/80 and CD11c mRNA expression levels in the visceral adipose tissue (VAT) were also decreased by the RS2 and RS4 treatments in both GF and CVZ mice, but these reductions did not consistently reach statistical significance (Fig. 6c, d). CD11c mRNA expression in SAT and VAT was strongly correlated with the index of insulin resistance for both CVZ and GF mice (Pearson *r* = 0.75 and 0.68 in SAT, Pearson *r* = 0.71 and 0.74 in VAT, *p* < 0.0001).

To confirm our observations that RS feeding is associated with a modulation of ATM, we isolated the stromal vascular fraction (SVF) from the SAT of CVZ mice. As expected, feeding a WD significantly increased the abundance of subcutaneous ATM as well as the ratio of M1 (F4/80^+^ CD11b^+^ CD11c^+^) to M2 (F4/80^+^ CD11b^+^ CD206^+^ or F4/80^+^ CD11b^+^ CD301^+^) macrophages (Fig. 6e–h; Additional file 5: Fig. S4a, b). Dietary supplementation with RS2 or RS4 significantly reduced the percentage of ATM in the SVF (Fig. 6e). Of interest, feeding RS2 and RS4 reduced both the M1 and M2 macrophage populations (Fig. 6f–h; Additional file 5: Fig. S4a, b). Plasma and SAT mRNA expression levels of monocyte chemoattractant protein 1 (MCP-1; also known as CCL2), a chemokine previously shown to affect macrophage infiltration into adipose tissues [48, 49], were not affected by RS feeding (Additional file 5: Fig S4c, d), suggesting that changes in this particular chemokine are not responsible for the decreased subcutaneous ATM accumulation observed when mice are fed RS. All together, these data demonstrate that RS-mediated improvements in insulin sensitivity are associated with a reduced number of both M1 and M2 macrophages.

**Fig. 7:**
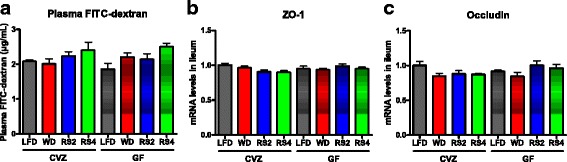
Markers of intestinal permeability in germ-free (*GF*) and conventionalized (*CVZ*) C3H mice fed experimental diets for 8 weeks. **a** Gut permeability as assessed by administering 4 kDa FITC-dextran. **b** Zonula occludens 1 (ZO-1) and **c** occludin mRNA expression in the ileum. *LFD* low-fat diet, *WD* Western diet, *RS2* WD with resistant starch 2, *RS4* WD with resistant starch 4. Mean ± SEM. **a** *N* = 6–8. **b**–**c** *N* = 7–8

Changes in adipose tissue physiology and reduced liver and adipose tissue macrophage infiltration in obese mice have been associated with an improvement in gut permeability [50–52]. To that end, we investigated intestinal permeability by administering 4 kDa FITC-dextran to GF and CVZ mice but found that none of the dietary treatments significantly altered gut permeability during the fasting state (Fig. 7a). In accordance with this observation, ileal expression of ZO-1 and occludin were also not affected by any of the experimental diets (Fig. 7b, c). Altogether, these findings suggest that RS decrease expression of adipose tissue macrophage markers independently of the gut microbiota and changes in gut permeability.

### Resistant starches redressed Western diet-induced changes in cecal bile acid profiles in both germ-free and conventionalized mice

Several studies report changes in the bile acid (BA) pool composition of individuals diagnosed as prediabetic or with type 2 diabetes [53–57]. BAs are capable of regulating both insulin sensitivity and glucose homeostasis as well as reducing the inflammatory activity of macrophages via nuclear farnesoid X receptor (FXR) and membrane-bound TGR5 signaling [45, 58, 59]. Moreover, RS feeding has been shown to alter BA levels in both humans and rodents [60–62]. These reports prompted us to analyze the cecal BA pool of GF and CVZ mice fed RS.

**Fig. 8:**
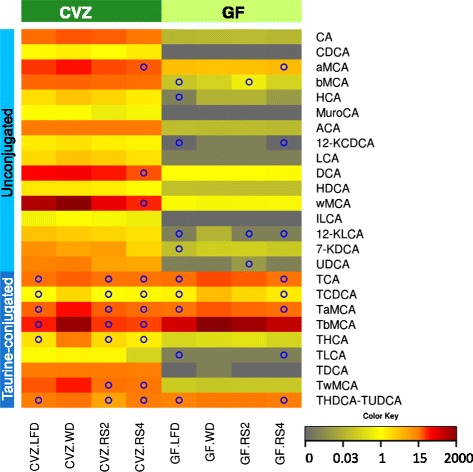
Resistant starches restored the cecal bile acid profiles of germ-free (*GF*) and conventionalized (*CVZ*) C3H mice fed experimental diets for 8 weeks. Mean bile acid concentration for each dietary treatment (nmol/g of cecal content). Only bile acids with mean concentrations above 1 nmol/g for at least one experimental treatment are shown. *LFD* low-fat diet, *WD* Western diet, *RS2* WD with resistant starch 2, *RS4* WD with resistant starch 4, *CA* cholic acid, *CDCA* chenodeoxycholic acid, *MCA* muricholic acid, *HCA* hyocholic acid, *MuroCA* murocholic acid, *ACA* allocholic acid, *LCA* lithocholic acid, *DCA* deoxycholic acid, *HDCA* hyodeoxycholic acid, *ILCA* isolitocholic acid, *UDCA* ursodeoxycholic acid, *K* keto derivative, *T* taurine-conjugated species. Complete abbreviation list is available in the supplemental materials and methods. °*p* < 0.05 versus WD fed mice of the same microbial status (one-way ANOVA with Dunnett’s post hoc tests after normalization by log-transformation when needed). *N* = 7–8

The cecal BA pool was markedly different between GF and CVZ mice, consistent with previous findings [63]. Specifically, profiles from GF mice were dominated by taurine-conjugated primary bile acids (including tauro-β-muricholic acid) whereas the patterns from CVZ mice were more complex and included elevated levels of secondary BAs (Fig. 8, Additional file 3: Table S3, and Additional file 5: Figure S4e, f). WD feeding significantly increased the concentration of several BAs, such as taurocholic acid (TCA) and taurochenodeoxycholic acid (TCDCA), in both GF and CVZ mice while supplementing the WD with RS redressed these alterations in a microbiota-independent manner (Fig. 8). Interestingly, we observed strong correlations between TCA and TCDCA concentrations and plasma insulin levels, the index of insulin resistance, and ATM markers (*q* values below 0.0005 for insulin and index of insulin resistance, Additional file 3: Table S3). Together, these findings indicate that RS feeding impacts the cecal BA pool—especially taurine-conjugated BA—in a microbiota-independent manner.

## Discussion

An understanding of how dietary compounds such as RS can modify risk factors for complex diseases provides an important basis for the rational development of nutritional strategies for disease prevention and treatment. The gut microbiota is generally assumed to mediate RS-associated health benefits by fermenting RS to SCFA, which have numerous known effects on host physiology [64]. Consistent with this view, studies in rodents implicate bacterial fermentation as a mediator of GLP-1 and PYY secretion in bringing about the metabolic benefits of RS [32]. Indeed, GLP-1 and PYY are pleotropic, gut-derived hormones that, among others, affect glucose homeostasis and appetite [18]. Because microbial responses to fiber and other prebiotics can be highly individualized [33, 65], determining how—and even if—the gut microbiota contributes to the physiological effects is an important step toward the development of efficient nutritional strategies. Although our findings confirm the pronounced effect of RS on gut microbiota composition and structure [33, 66], they do not support a causative role for these shifts in RS-induced metabolic improvements, including insulin sensitivity. Indeed, feeding RS2 and RS4 resulted in vastly different effects on the composition of the gut microbiota; however, both RS types induced physiological changes that were highly similar (with RS4 more consistently improving metabolic parameters as compared to RS2). Together, these findings further imply the importance of a microbiota-independent pathway in RS-mediated metabolic benefits.

Remarkably, the physiological characteristics of this microbiota-independent pathway in C3H mice, which include improvements in insulin sensitivity without effects on body weight, fat mass, and gut hormone levels, mirror those observed clinically in humans where improvement in insulin sensitivity is the primary beneficial outcome of feeding RS with little to no effect on body composition [8–11]. Moreover, these RS-mediated improvements in insulin sensitivity in humans are also observed without a concomitant increase in fasting GLP-1 levels [67]. Clearly, we cannot exclude the possibility that the improvements in insulin sensitivity conferred by RS consumption occur via different mechanisms in mice versus humans. However, similar metabolic effects were observed both during human RS feeding studies and when RS was fed to mice in the absence of a microbiota. Together, these findings indicate that the most important clinical improvement induced by dietary RS in humans may occur without a contribution from the gut microbiota, bacterial fermentation, and gut hormones such as GLP-1 and PYY.

In contrast to our findings and those from human clinical trials, reductions in fat mass and/or body weight are often observed in rodent RS feeding studies in addition to the improvements in insulin resistance [14]. These improvements in adiposity require bacterial fermentation in the gut and are connected with increases in gut hormones GLP-1 and PYY [32, 68, 69]. This discrepancy can potentially be explained by a RS dose-dependent effect. Rodent studies showing an effect of RS on body weight and fat mass provided doses of 30% to 55% (*w*/*w*) [32, 68, 70, 71], which are substantially higher than the doses used in the present study (10% RS) and in human clinical trials [8–11]. Feeding a lower dose of RS might account for the absence of an RS-mediated effect on GLP-1 and PYY levels and the lack of improvement in weight and adiposity. Indeed, secretion of GLP-1 and PYY into the intestine is stimulated by SCFA [17, 18], which are, as metabolic end products of bacterial fermentation, produced in a substrate-dependent manner. However, the doses of RS required to induce fat loss in rodents are not realistic for incorporation into human diets, leading one to question the clinical applicability of RS for controlling adiposity. Nonetheless, diets containing lower doses of RS represent an exciting opportunity to improve insulin sensitivity.

In addition to feeding lower RS doses to be more consistent with human studies, we also formulated mouse diets in such a way as to facilitate comparison between a diet with native starch to a diet where a portion of the native starch was replaced with RS. Although this approach does create differences in caloric content among the diets, it is utilized extensively in human nutrition and eliminates concerns over using so-called inert fibers (such as cellulose) to control for energy density that may actually exert their own effects on the host [14, 33, 38, 39, 72, 73]. As no significant effects of RS were observed on energy intake, body weight, body weight gain, or adipose tissue weights in the current study, the observed metabolic improvements likely occur independently of changes in energy intake and fat accumulation. We do acknowledge that a lack of caloric equivalence among diets can be considered a study limitation. Additionally, another limitation of our work is the absence of a functional assessment of glucose homeostasis in germ-free mice fed RS. In place of this assay, we instead measured a hallmark of insulin sensitivity (HOMA-IR), which also allowed us to simultaneously measure several additional metabolic parameters. Certainly, performing such functional experiments (e.g., insulin and oral glucose tolerance tests) would provide additional, valuable mechanistic insights.

Upon observing RS-induced improvements in insulin sensitivity in mice independently of the gut microbiota, we explored potential explanations for how the beneficial effects of RS may occur without a microbial contribution. One such possibility is that RS regulate innate immunity. Numerous studies have implicated CD11c^+^ ATMs in the development of insulin resistance [4], and RS-mediated improvements in insulin sensitivity in rats have been associated with reduced CD11c expression in adipose tissue [70]. In our study, we demonstrate a strong correlation between ATM marker expression and the index of insulin resistance in both GF and CVZ animals. We recognize that evaluating immune and metabolic parameters in germ-free mice has its inherent limitations [35, 74–76], and we cannot exclude the possibility that the immature immune system present in germ-free mice was not fully restored by the conventionalization process. Nevertheless, we did observe decreases in ATM markers in both germ-free and conventionalized mice fed RS that were consistent with previous findings obtained in immunocompetent rats [70]. Although the exact mechanism by which RS improves insulin levels and influences ATM abundance in the absence of a microbiota remains unclear, our findings suggest that RS-induced modulation of the BA pool may be involved. Indeed, connections among BA signaling, insulin sensitivity, and macrophages are emerging. Of note, deletion of the BA membrane receptor *Tgr5* specifically in macrophages increased ATM accumulation and aggravated insulin resistance in obese animals [59]. Considering our observations that feeding an RS-supplemented diet redressed the concentrations of BA elevated by a WD and the strong correlations between BAs, macrophage markers, and insulin metabolism, it is tempting to speculate that RS-induced modulation of the BA profile reduces macrophage migration, thereby contributing to improvements in insulin sensitivity. Clearly, future mechanistic studies are necessary to determine how RS improves insulin resistance independently of the gut microbiota.

## Conclusions

In conclusion, this study unequivocally demonstrates that the gut microbiota is not required to bring about all the metabolic benefits that arise from RS consumption in mice. Instead of relying on correlations between physiological parameters and specific microbial taxa, which is common practice but cannot assign causality, we experimentally determined the causative role of the gut microbiota using germ-free mice. We argue that similar gnotobiotic animal research could constitute an essential component to determining the prebiotic properties of a specific dietary compound [22]. Based on our findings and those in the literature [32], we propose a model in which RS induces health benefits via both microbiota-dependent and microbiota-independent pathways. When RS are fed at higher doses, metabolites from bacterial fermentation are released at physiologically relevant levels, leading to a decrease in body weight and/or fat mass via a GLP1-PYY-dependent mechanism [32]. When fed in clinically relevant doses, RS improves insulin resistance independently of the gut microbiota, possibly altering bile acid signaling as well as adipose tissue immune modulation. Although our findings are supported by observations in humans showing an impact of RS feeding on insulin sensitivity without any effect on fat mass or increase in fasting GLP-1 levels [8–10, 40, 67], additional studies are necessary to elucidate the exact mechanisms, dose-response relationships, and the role of microbiota variability in both humans and animal cohorts.

## Methods

### Mice

Germ-free (GF) C3H and C57BL/6 (B6) male mice were born and reared in flexible film isolators and maintained under gnotobiotic conditions at the University of Nebraska-Lincoln (UNL). Conventionalized (CVZ) mice were used for all experiments (unless specified otherwise) to limit genetic, maternal, and early-life-related confounding factors when comparing to GF animals.

Experimental diets were introduced to both GF and CVZ mice 9 and 21 days after conventionalization of C3H and B6 mice, respectively. Body weight was monitored weekly after conventionalization. Food intake was also monitored weekly after introduction of the experimental diets, but multiple measurements were hampered by spillage issues due to softness and crumbling of the Western diet. Feces were collected on the day of necropsy. The Institutional Animal Care and Use Committee at the University of Nebraska-Lincoln approved all procedures involving animals.

Germ-free (GF) status of all experimental mice was confirmed at least twice (prior to introducing the experimental diet and during the last week of the experiment) by analyzing fresh feces via PCR using universal bacterial primers targeting the 16S rRNA gene (30 cycles, primers 8F and 1391R) [77] and aerobic and anaerobic culture in brain heart infusion, Wilkins-Chalgren, and yeast mold broths for 7 days at 37 °C. Conventionally raised (CONV) B6 male mice were obtained from Jackson Laboratory (Bar Harbor, ME). Conventionalized (CVZ), CONV, and GF mice were housed in the same room on autoclaved bedding and fed the same autoclaved water and diet. All mice within an experiment were born within 5 to 9 days of one another. Three to ten mice were obtained per litter, and littermates were assigned to cages of two to three mice each. Mice were then randomly assigned to each treatment/intervention based on body weight at the time of conventionalization and diet introduction. No differences in body weight variance were observed among the treatment groups on the day of randomization. Conventionalization, insulin tolerance tests, and gut permeability assays are described in Additional file 6: supplemental methods.

### Diets

Mice were fed an autoclavable chow diet (LabDiet 5K67, Purina Foods, St. Louis, MO) after weaning and during the period of time between conventionalization and introduction of the experimental diets. Experimental diets were prepared by Research Diets (New Brunswick, NJ) and sterilized by γ-irradiation (min 50 kGy, Neutron Products, Dickerson, MD). Irradiation efficacy was assessed by testing 50 spore strips of *Bacillus pumillus* (NAMSA, Northwood, OH) placed between bags of diet before irradiation and then incubated after irradiation in Soybean-Casein Digest broth at 33 °C for 7 days. Mice were fed a LFD (D12450K) and a customized WD (45% kcal from fat and 17% kcal from sucrose with low maltodextrine/high starch compared to D12451) where part of the corn starch was replaced by either 10% RS type 2 (RS2; HI-MAIZE® 260, Ingredion Incorporated, Westchester, IL) or 10% RS type 4 (RS4; Fibersym® RW, MGP Ingredients, Atchinson, KS). HI-MAIZE® 260 is derived from high amylose corn starch; it has a caloric content of 1.5 kcal/g and contains 62.50% of RS. Fibersym® RW is a chemically modified phosphorylated cross-linked RS4 prepared from wheat starch [78]; it has a caloric content of 0.44 kcal/g and contains 89.10% of RS. Diet composition is summarized in Table 1, with full details provided in Additional file 3: Table S4.

### Blood and tissue analyses

Mice were euthanized via carbon dioxide asphyxiation. Blood was collected into EDTA-containing tubes and centrifuged (13,000×*g*, 3 min, 4 °C). At the time of collection, blood samples were treated with a DPPIV inhibitor (Millipore, Billerica, MA) and a protease inhibitor cocktail (Sigma, Saint-Louis, MO). Plasma insulin was measured using an ELISA-based assay (Mercodia, Uppsala, Sweden). The index of insulin resistance (IR), also called HOMA-IR, was calculated based on the original model from Matthews et al.: fasting glucose (mg/dL) × fasting insulin (mU/L)/405. For reference, HOMA-IR values in healthy humans are equal to 1, whereas IR values for healthy mice are greater than one [79–82]. Percent change values were calculated to assist the reader in understanding relative changes in the IR between each treatment compared to the WD control. Gut peptides, gut hormones, MCP-1, and adiponectin were measured using a Mouse Metabolic Magnetic Bead Panel or a Mouse Adiponectin Single Plex Magnetic Bead Kit (Milliplex, Millipore, Billerica, MA) and a MAGPIX instrument (Luminex Corporation, Austin, TX). Gene expression and flow cytometric analyses are described in Additional file 6: supplemental methods.

### Microbial community analysis

Gut microbiota composition was assessed by 16S rRNA gene sequencing of fecal samples. Bacterial DNA was extracted from feces using the QIAamp DNA Stool Mini Kit (Qiagen, Valencia, CA) with a bead-beating step as previously described [33]. Amplicon sequencing of the fecal microbiota was performed at the University of Minnesota Genomics Center as described in Additional file 6: supplemental methods.

Initial quality filtering of the reads was performed with the Illumina Software, yielding an average of 53,426 filter-passed reads per sample (accession numbers provided in supplemental methods). Quality scores were visualized with the FastQC software (http://www.bioinformatics.babraham.ac.uk/publications.html), and reads were trimmed to 250 bp (R1) and 230 bp (R2) with the FASTX-Toolkit (http://hannonlab.cshl.edu/fastx_toolkit). Next, reads were merged with the merge-illumina-pairs application (with *p* value = 0.02, enforced Q30 check, perfect matching to primers which are removed by the software, and otherwise default settings including no ambiguous nucleotides allowed) [83]. For samples with >20,000 merged reads, a subset of 20,000 reads was randomly selected using Mothur 1.32.1 centos 5.5 for Linux [84] to avoid large disparities in the number of sequences. Subsequently, the UPARSE pipeline implemented in USEARCH v7.0.1001 [85] was used to further process the sequences. Putative chimeras were identified against the Gold reference database and removed. Clustering was performed with 98% similarity cutoff to designate operational taxonomic units (OTUs). Non-chimeric sequences were also subjected to taxonomic classification using the RDP MultiClassifier 1.1 from the Ribosomal Database Project [86] for phylum to genus characterization of the fecal microbiota. The phylotypes were computed as percent proportions based on the total number of sequences in each sample. Alpha and beta diversity indexes were calculated using QIIME [87]. PCoA plot of the beta-diversity indexes were obtained using EMPeror [88]. We used LEfSe to calculate and visualize the LDA Effect size [89] (http://huttenhower.sph.harvard.edu/galaxy/).

### Bile acid analysis

Forty-six bile acids were quantified in cecal contents by ultrahigh performance liquid chromatography—multiple-reaction monitoring mass spectrometry (UPLC-MRM-MS) at the University of Victoria Genome British Columbia Proteomics Centre using a protocol adapted from Han et al. [90] (see Additional file 6: supplemental methods).

### Statistical analyses

A sample size of *n* = 8 was calculated for the four treatment groups after considering a power of 80%, a significance level of 0.05, an effect size of 0.7, and a 10% attrition rate using G*Power 3.1.9.2 [91]. Data were analyzed using Prism 5.0 (GraphPad Software, San Diego, CA) via a one-way ANOVA followed by Dunnett’s pairwise comparison post hoc test with the WD group as control. The global effects of microbial status (presence or absence of the gut microbiota) and dietary intervention were assessed using two-way ANOVA. All data were checked for normality using tests available in Prism 5.0 (Kolmogorov-Smirnov, D’Agostino and Pearson, and Shapiro-Wilk normality tests). Data determined to be non-normal even after log-transformation were analyzed using a Kruskal-Wallis test and Dunn’s post-tests as indicated in the legend. Body weight evolution was analyzed by two-way ANOVA with repeated measures followed by a Bonferroni post hoc test (Fig. 1). For parametric analyses of the gut microbiota, the *p* value of the one-way ANOVA was adjusted to control the false discovery rate for multiple tests according to the Benjamini and Hochberg procedure [92]. Robust (Huber) estimation was used to weigh down outliers, as implemented in JMP Pro 11 (SAS Institute, Cary, NC). R software (R Foundation for Statistical Computing, Vienna, Austria) [93] was used for multiple correlation analyses and *p* value adjustments according to the Benjamini and Hochberg procedure. *p* < 0.05 was considered statistically significant.

## Additional files

Additional file 1: Figure S1. Resistant starches improved insulin sensitivity in B6 mice in the presence of a microbiota. Glycemic response after insulin injection in conventional 16 week-old B6 mice fed experimental diets for 7 weeks (a, b) (LFD, low fat diet; WD, Western diet; RS2, WD with resistant starch 2; RS4, WD with resistant starch 4). Body weights at necropsy (c), body weight gains over 8 weeks of dietary intervention (d), subcutaneous adipose tissue (SAT) weights at necropsy (e) and energy intake (as recorded over a week at week 4) (f) in conventional B6 mice fed experimental diets for 8 weeks. Although RS4 feeding tended to reduce the body and subcutaneous adipose tissue weights of B6 mice, these differences were not statistically significant (p = 0.18 and p = 0.51, respectively, RS4 versus WD according to Dunnett’s post-hoc tests). #p=0.08, *p<0.05, ***p<0.001 versus WD (one-way ANOVA with Dunnett’s post-hoc tests). Mean ± SEM. (a, b) N=5-9; (c-e) N=8; (f) N=4 cages. (PDF 30 kb)
Additional file 2: Figure S2. Feeding resistant starches changed the gut microbiota composition. (a) Principal Coordinate Analysis plot of β-diversity based on Bray-Curtis distance. (b) Principal Coordinate Analysis plot of β-diversity based on Unweighted Unifrac distance. (c) LEfSe cladogram highlighting the bacterial taxa specifically enriched in each dietary group. (d) LEfSe results highlighting the bacterial taxa specifically enriched in each dietary group. LFD, low fat diet; WD, Western diet; RS2, WD with resistant starch 2; RS4, WD with resistant starch 4. N=8. (PDF 1390 kb)
Additional file 3: Supplemental Tables. List of OTUs significantly affected by dietary intervention. Each OTU is identified at the species level and the percentage of homology is indicated (%ID). R_FDR_p indicates the p-value of the one-way ANOVA adjusted to control the false discovery rate for multiple tests according to the Benjamini and Hochberg procedure and with Robust (Huber) estimation to down weight outliers. LFD, WD, RS2, RS4 represent the mean relative abundance of each OTU (as a fraction of one) for each group; SEM are indicated in the last 4 columns. *p<0.05 versus WD (one-way ANOVA with Dunnett’s post-hoc tests). N=8. **Table S2.** List of bacterial taxa significantly affected by dietary intervention. R_FDR_p indicates the p-value of the one-way ANOVA adjusted to control the false discovery rate for multiple tests according to the Benjamini and Hochberg procedure and with Robust (Huber) estimation to down weight outliers. LFD, WD, RS2, RS4 represent the mean relative abundance of each bacterial taxon (as a fraction of one) for each group; SEM are indicated in the last 4 columns. *p<0.05 versus WD (one-way ANOVA with Dunnett’s post-tests). N=8. **Table S3. **Bile acid dataset summary table, including correlations between bile acids and selected host physiological parameters. Mean concentrations (in nmole/g of cecal contents) with SEM for each group along with fold changes. Pearson r, and p- and q-values for the Pearson correlation tests are presented. Only bile acids with mean concentrations above 1nmole/g for at least one experimental treatment were included in the multiple correlation analyses. Correlations with a p-value <0.05 are highlighted in pink and correlations with a p- and a q-value <0.05 are highlighted in red. N=6-8/group. **Table S4. **Diet composition. Ingredient quantity, energy density and macronutrient repartition for all the experimental diets. LFD, low fat diet; WD, Western diet; RS2, WD with resistant starch 2; RS4, WD with resistant starch 4. References to the standard diet formulations from Research Diets are also included. Mice were fed a low fat diet (LFD, D12450K) and a customized Western diet (WD, 45% kcal from fat and 17% kcal from sucrose with low maltodextrine/high starch compared to D12451) where a portion of the corn starch was replaced by either 10% RS type 2 (RS2; HI-MAIZE® 260, Ingredion Incorporated, Westchester, IL) or 10% RS type 4 (RS4; Fibersym® RW, MGP Ingredients, Atchinson, KS). HI-MAIZE® 260 is derived from high amylose corn starch; it has a caloric content of 1.5 kcal/g and it contains 62.5% RS. Fibersym® RW is a chemically modified phosphorylated cross-linked RS4 prepared from wheat starch; it has a caloric content of 0.44 kcal/g and contains 89.1% RS. (PDF 260 kb)
Additional file 4: Figure S3. Resistant starches did not impact body weights, white adipose tissue weights, tibialis muscle weights and plasma leptin levels in germ-free (GF) or conventionalized (CVZ) C3H mice fed experimental diets for 8 weeks. (a, b, d) Body, adipose tissue and tibialis weights. (c) Plasma leptin levels. *p<0.05 versus WD fed mice of the same microbial status (one-way ANOVA with Dunnett’s post-hoc tests). For C, GF-RS4 data were determined to be non-normal even after log-transformation and were analyzed using a Kruskal-Wallis test then Dunn’s post-tests. LFD, low fat diet; WD, Western diet; RS2, WD with resistant starch 2; RS4, WD with resistant starch 4. Mean ± SEM. N=7-8. (PDF 29 kb)
Additional file 5: Figure S4. Resistant starches modulated subcutaneous adipose tissue macrophage accumulation and cecal bile acid profiles in C3H mice fed experimental diets for 8 weeks. Two different markers were used to identify M2 macrophages in C3H mice, CD206 and CD301. Both markers provided similar results, as shown in Fig. 5 for CD206 and Fig S5 for CD301. (a) Percentage of M2 (CD301+) macrophages in the stromal vascular fraction (SVF) of the SAT of C3H mice. (b) Ratio of M1/M2, using CD301 as a M2 marker. (c) Plasma monocyte-chemoattractant protein-1 (MCP-1) levels. (d) MCP-1 expression in subcutaneous adipose tissue (SAT). (e) Principal Component Analysis plot of the bile acid profiles by microbial status. (f) Principal Component Analysis plot of the bile acid profiles by microbial status and dietary treatment. CVZ: conventionalized. GF: germ-free. LFD, low fat diet; WD, Western diet; RS2, WD with resistant starch 2; RS4, WD with resistant starch 4. #p=0.07, *p<0.05 vs WD fed mice of the same microbial status (one-way ANOVA with Dunnett’s post-hoc tests). Microbial status significantly impacted MCP-1 mRNA expression in the SAT (analysis of all 8 treatments using two-way ANOVA). (a-d) Mean ± SEM. (a, b) N=14-15 except for the LFD where N=6. (c, d) N=6-8. (e, f) N=7-8. (PDF 98 kb)
Additional file 6: Supplemental materials and methods. (PDF 208 kb)

## Abbreviations

ACA: Allocholic acid
ATM: Adipose tissue macrophage
B6: C57BL/6
BA: Bile acid
C3H: C3H/HeN
CA: Cholic acid
CDCA: Chenodeoxycholic acid
CONV: Conventionally raised
CVZ: Conventionalized
DCA: Deoxycholic acid
DIO: Diet-induced obesity
GF: Germ-free
HCA: Hyocholic acid
HDCA: Hyodeoxycholic acid
ILCA: Isolitocholic acid
K: Keto derivative
LCA: Lithocholic acid
LFD: Low-fat diet
MCA: Muricholic acid
MuroCA: Murocholic acid
RS: Resistant starch
RS2: Resistant starch type 2
RS4: Resistant starch type 4
SAT: Subcutaneous adipose tissue
SCFA: Short-chain fatty acids
SVF: Stromal vascular fraction
T: Taurine derivative
UDCA: Ursodeoxycholic acid
VAT: Visceral adipose tissue
WD: Western diet
