# Supplementary figures and images for: Resistant starch can improve insulin sensitivity independently of the gut microbiota
Source: Microbiome. 2017 Feb 7;5:12. doi: 10.1186/s40168-017-0230-5 (PMC5294823; doi:10.1186/s40168-017-0230-5)

Figure S1

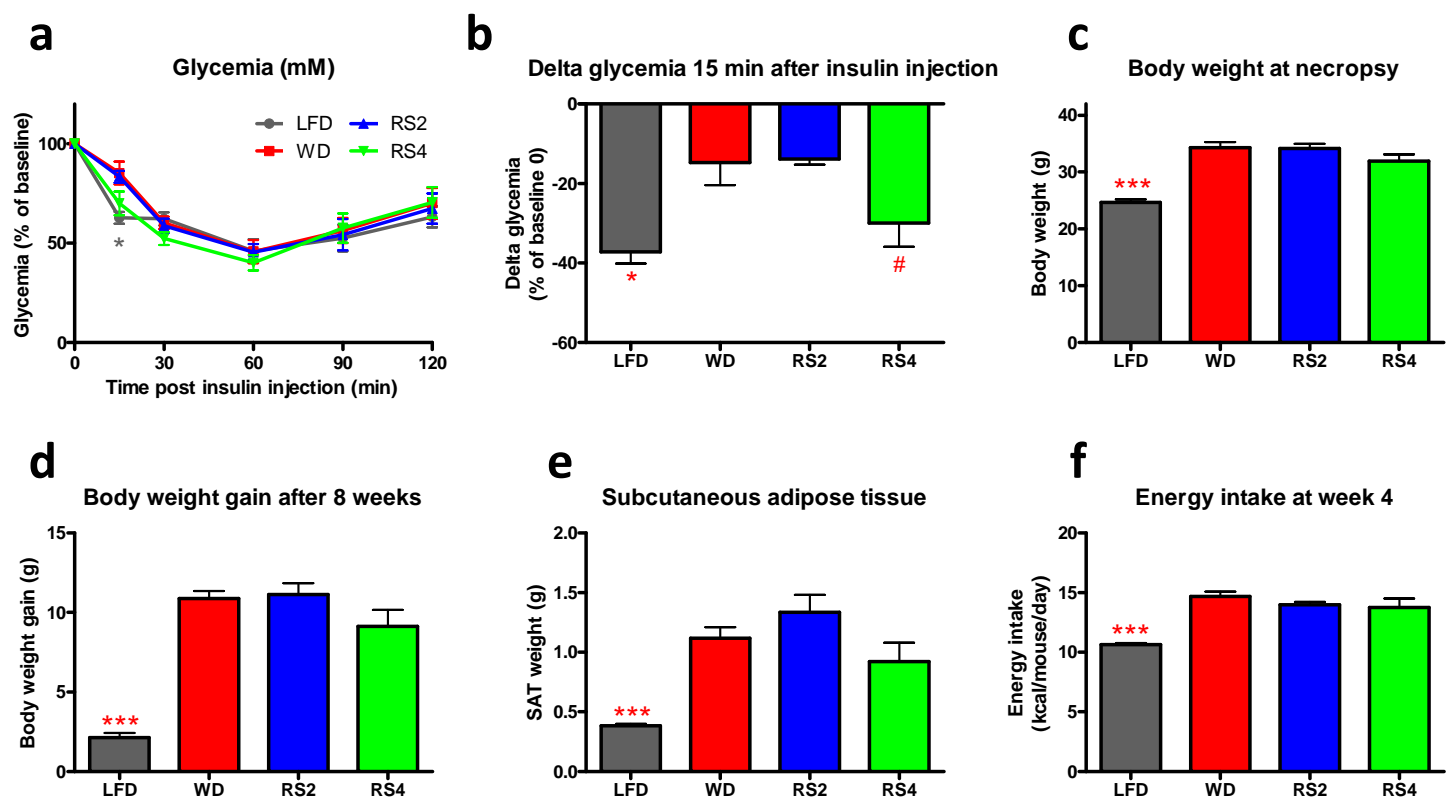

Supplement: Additional file 1: Figure S1. — Resistant starches improved insulin sensitivity in B6 mice in the presence of a microbiota. Glycemic response after insulin injection in conventional 16 week-old B6 mice fed experimental diets for 7 weeks (a, b) (LFD, low fat diet; WD, Western diet; RS2, WD with resistant starch 2; RS4, WD with resistant starch 4). Body weights at necropsy (c), body weight gains over 8 weeks of dietary intervention (d), subcutaneous adipose tissue (SAT) weights at necropsy (e) and energy intake (as recorded over a week at week 4) (f) in conventional B6 mice fed experimental diets for 8 weeks. Although RS4 feeding tended to reduce the body and subcutaneous adipose tissue weights of B6 mice, these differences were not statistically significant (p = 0.18 and p = 0.51, respectively, RS4 versus WD according to Dunnett’s post-hoc tests). #p=0.08, *p<0.05, ***p<0.001 versus WD (one-way ANOVA with Dunnett’s post-hoc tests). Mean ± SEM. (a, b) N=5-9; (c-e) N=8; (f) N=4 cages. (PDF 30 kb) [file 40168_2017_230_MOESM1_ESM.pdf]

Figure S2

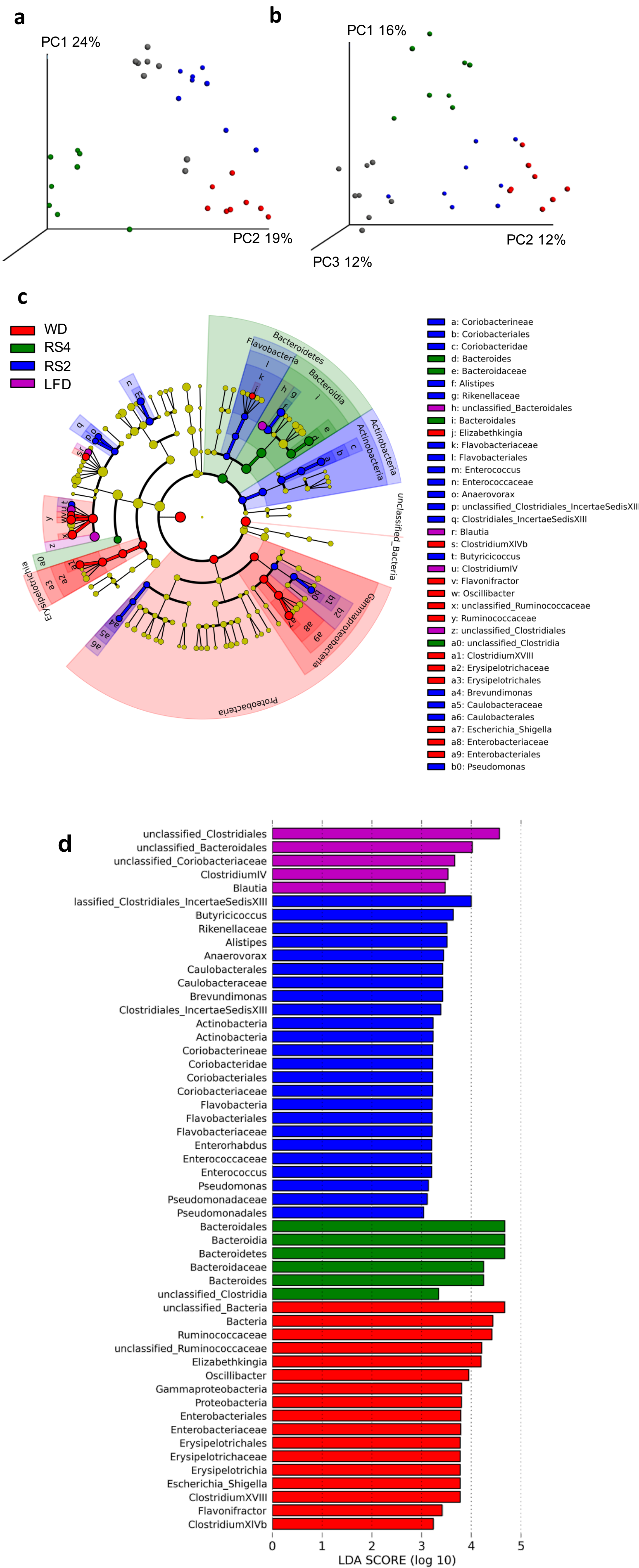

Supplement: Additional file 2: Figure S2. — Feeding resistant starches changed the gut microbiota composition. (a) Principal Coordinate Analysis plot of β-diversity based on Bray-Curtis distance. (b) Principal Coordinate Analysis plot of β-diversity based on Unweighted Unifrac distance. (c) LEfSe cladogram highlighting the bacterial taxa specifically enriched in each dietary group. (d) LEfSe results highlighting the bacterial taxa specifically enriched in each dietary group. LFD, low fat diet; WD, Western diet; RS2, WD with resistant starch 2; RS4, WD with resistant starch 4. N=8. (PDF 1390 kb) [file 40168_2017_230_MOESM2_ESM.pdf]

Figure S3

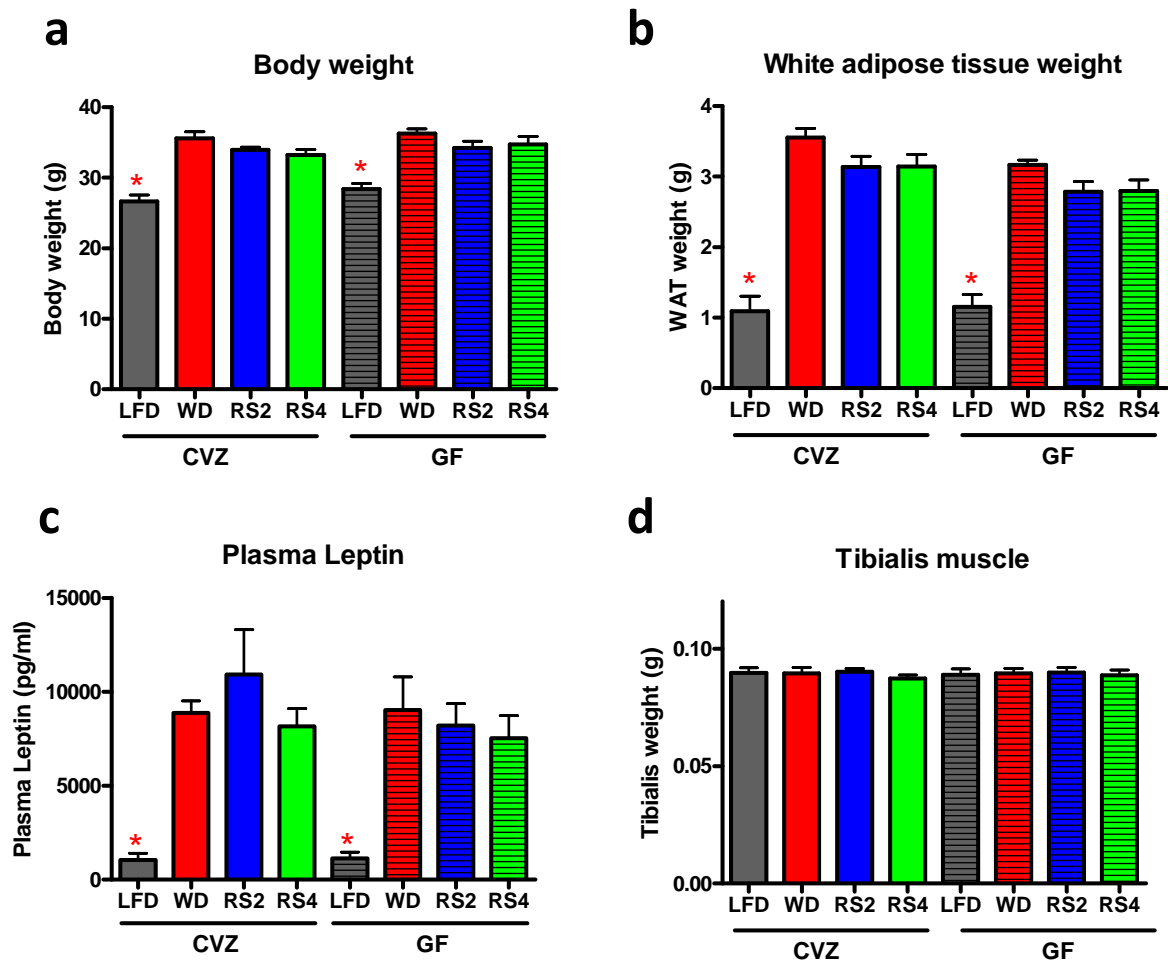

Supplement: Additional file 4: Figure S3. — Resistant starches did not impact body weights, white adipose tissue weights, tibialis muscle weights and plasma leptin levels in germ-free (GF) or conventionalized (CVZ) C3H mice fed experimental diets for 8 weeks. (a, b, d) Body, adipose tissue and tibialis weights. (c) Plasma leptin levels. *p<0.05 versus WD fed mice of the same microbial status (one-way ANOVA with Dunnett’s post-hoc tests). For C, GF-RS4 data were determined to be non-normal even after log-transformation and were analyzed using a Kruskal-Wallis test then Dunn’s post-tests. LFD, low fat diet; WD, Western diet; RS2, WD with resistant starch 2; RS4, WD with resistant starch 4. Mean ± SEM. N=7-8. (PDF 29 kb) [file 40168_2017_230_MOESM4_ESM.pdf]

Figure S4

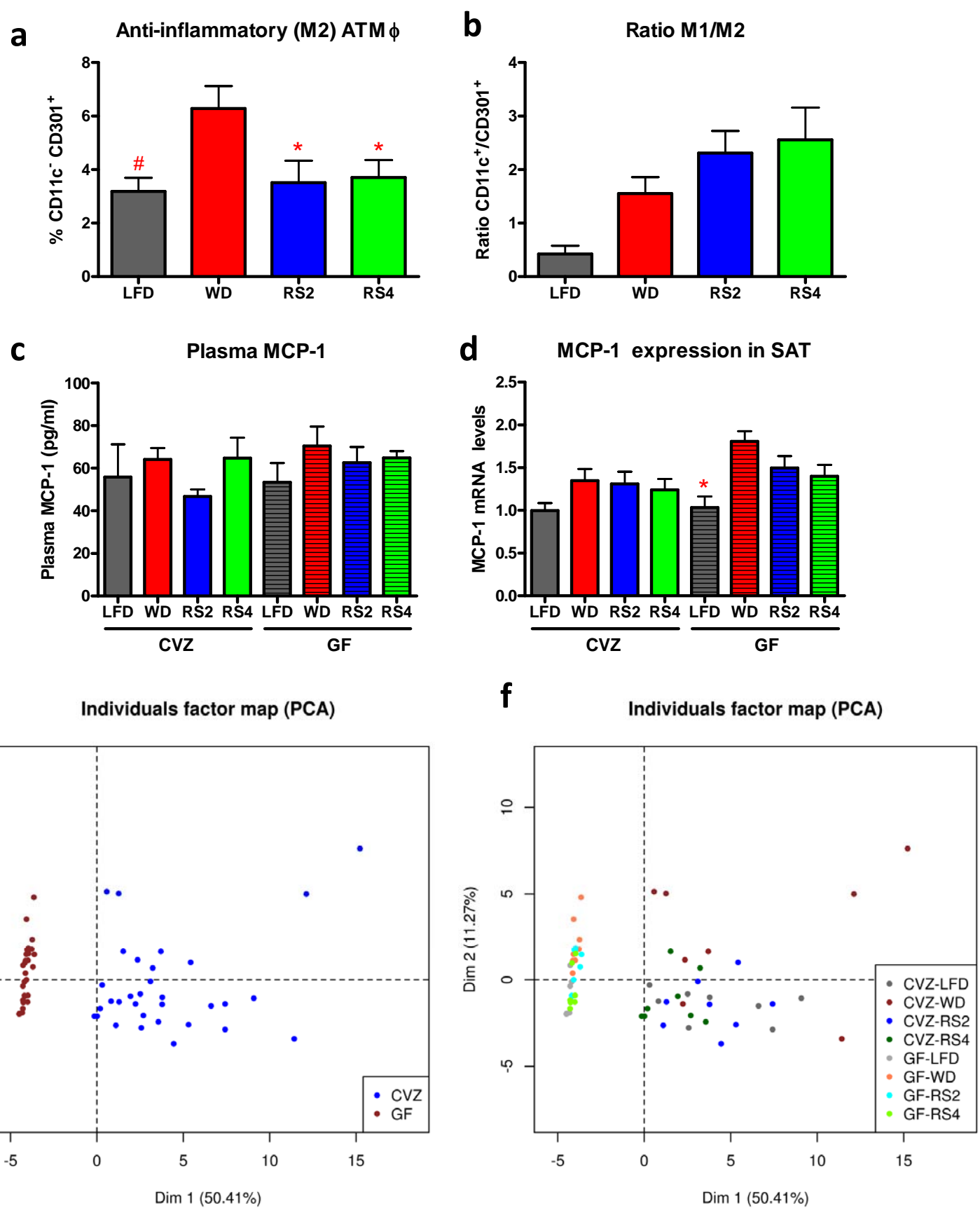

Supplement: Additional file 5: Figure S4. — Resistant starches modulated subcutaneous adipose tissue macrophage accumulation and cecal bile acid profiles in C3H mice fed experimental diets for 8 weeks. Two different markers were used to identify M2 macrophages in C3H mice, CD206 and CD301. Both markers provided similar results, as shown in Fig. 5 for CD206 and Fig S5 for CD301. (a) Percentage of M2 (CD301+) macrophages in the stromal vascular fraction (SVF) of the SAT of C3H mice. (b) Ratio of M1/M2, using CD301 as a M2 marker. (c) Plasma monocyte-chemoattractant protein-1 (MCP-1) levels. (d) MCP-1 expression in subcutaneous adipose tissue (SAT). (e) Principal Component Analysis plot of the bile acid profiles by microbial status. (f) Principal Component Analysis plot of the bile acid profiles by microbial status and dietary treatment. CVZ: conventionalized. GF: germ-free. LFD, low fat diet; WD, Western diet; RS2, WD with resistant starch 2; RS4, WD with resistant starch 4. #p=0.07, *p<0.05 vs WD fed mice of the same microbial status (one-way ANOVA with Dunnett’s post-hoc tests). Microbial status significantly impacted MCP-1 mRNA expression in the SAT (analysis of all 8 treatments using two-way ANOVA). (a-d) Mean ± SEM. (a, b) N=14-15 except for the LFD where N=6. (c, d) N=6-8. (e, f) N=7-8. (PDF 98 kb) [file 40168_2017_230_MOESM5_ESM.pdf]
