# Supplementary material for: Resistant starch can improve insulin sensitivity independently of the gut microbiota
Source: Microbiome. 2017 Feb 7;5:12. doi: 10.1186/s40168-017-0230-5 (PMC5294823; doi:10.1186/s40168-017-0230-5)
Supplement: Additional file 3: — Supplemental Tables. List of OTUs significantly affected by dietary intervention. Each OTU is identified at the species level and the percentage of homology is indicated (%ID). R_FDR_p indicates the p-value of the one-way ANOVA adjusted to control the false discovery rate for multiple tests according to the Benjamini and Hochberg procedure and with Robust (Huber) estimation to down weight outliers. LFD, WD, RS2, RS4 represent the mean relative abundance of each OTU (as a fraction of one) for each group; SEM are indicated in the last 4 columns. *p<0.05 versus WD (one-way ANOVA with Dunnett’s post-hoc tests). N=8. Table S2. List of bacterial taxa significantly affected by dietary intervention. R_FDR_p indicates the p-value of the one-way ANOVA adjusted to control the false discovery rate for multiple tests according to the Benjamini and Hochberg procedure and with Robust (Huber) estimation to down weight outliers. LFD, WD, RS2, RS4 represent the mean relative abundance of each bacterial taxon (as a fraction of one) for each group; SEM are indicated in the last 4 columns. *p<0.05 versus WD (one-way ANOVA with Dunnett’s post-tests). N=8. Table S3. Bile acid dataset summary table, including correlations between bile acids and selected host physiological parameters. Mean concentrations (in nmole/g of cecal contents) with SEM for each group along with fold changes. Pearson r, and p- and q-values for the Pearson correlation tests are presented. Only bile acids with mean concentrations above 1nmole/g for at least one experimental treatment were included in the multiple correlation analyses. Correlations with a p-value <0.05 are highlighted in pink and correlations with a p- and a q-value <0.05 are highlighted in red. N=6-8/group. Table S4. Diet composition. Ingredient quantity, energy density and macronutrient repartition for all the experimental diets. LFD, low fat diet; WD, Western diet; RS2, WD with resistant starch 2; RS4, WD with resistant starch 4. References to the st [file 40168_2017_230_MOESM3_ESM.pdf]

Supplemental Table 1

OTUid	family	% ID	R_FDR_p	LFD	WD	RS2	RS4	SEM LFD	SEM WD	SEM RS2	SEM RS4
OTU_37	unclassified Lachnospiraceae	1	4.05E-49	0.0055 *	0.0221	0.0023 *	0.0266	0.0024	0.0065	0.0009	0.0015
OTU_104	Clostridium sp.	0.943	2.18E-38	0.0007 *	0.0030	0.0045	0.0002 *	0.0001	0.0010	0.0003	0.0001
OTU_13	unclassified Lachnospiraceae	1	1.85E-22	0.0176 *	0.0000	0.0391 *	0.0017	0.0030	0.0000	0.0066	0.0011
OTU_80	unclassified Lachnospiraceae	1	5.98E-21	0.0008 *	0.0065	0.0093 *	0.0011 *	0.0002	0.0009	0.0009	0.0003
OTU_110	unclassified Lachnospiraceae	1	1.46E-20	0.0023	0.0017	0.0014	0.0000	0.0003	0.0004	0.0008	0.0000
OTU_43	Clostridiales bacterium CIEAF 0	0.963	3.94E-17	0.0165	0.0093	0.0007	0.0229 *	0.0050	0.0024	0.0002	0.0030
OTU_33	Clostridiales bacterium CIEAF 0	1	3.45E-15	0.0000	0.0006	0.0014	0.0473 *	0.0000	0.0002	0.0006	0.0069
OTU_138	Marvinbryantia sp.	1	2.57E-14	0.0000 *	0.0013	0.0019	0.0003 *	0.0000	0.0003	0.0005	0.0000
OTU_40	Clostridium sp. ASF502	1	8.92E-14	0.0151	0.0068	0.0123	0.0006	0.0032	0.0011	0.0033	0.0001
OTU_207	Lachnospiraceae sp.	0.924	5.65E-13	0.0233	0.0037	0.0145	0.0466 *	0.0026	0.0007	0.0070	0.0121
OTU_58	unclassified Lachnospiraceae	1	1.75E-12	0.0054	0.0032	0.0070	0.0001	0.0010	0.0016	0.0012	0.0001
OTU_27	unclassified Lachnospiraceae	1	6.30E-12	0.0100	0.0121	0.0098	0.0044 *	0.0017	0.0010	0.0022	0.0005
OTU_113	unclassified Lachnospiraceae	1	7.60E-12	0.0000 *	0.0008	0.0006	0.0005	0.0000	0.0002	0.0001	0.0001
OTU_21	unclassified Lachnospiraceae	0.975	7.60E-12	0.0186 *	0.0089	0.0043	0.0224 *	0.0031	0.0017	0.0011	0.0028
OTU_98	Clostridium XIVb sp.	0.971	9.74E-12	0.0025	0.0025	0.0019	0.0006 *	0.0004	0.0003	0.0003	0.0001
OTU_107	Clostridium XIVa sp.	1	3.74E-11	0.0003 *	0.0021	0.0024	0.0013	0.0002	0.0004	0.0003	0.0004
OTU_134	unclassified Lachnospiraceae	1	8.01E-11	0.0002	0.0001	0.0014 *	0.0013 *	0.0001	0.0000	0.0002	0.0002
OTU_115	unclassified Lachnospiraceae	1	1.72E-10	0.0020 *	0.0000	0.0002	0.0012 *	0.0005	0.0000	0.0001	0.0002
OTU_71	unclassified Lachnospiraceae	0.971	2.90E-10	0.0028	0.0054	0.0000 *	0.0043	0.0009	0.0018	0.0000	0.0008
OTU_16	Clostridium XIVa sp.	1	4.37E-10	0.0229	0.0246	0.0176	0.0067 *	0.0055	0.0028	0.0035	0.0011
OTU_240	Ruminococcus sp.	0.992	6.70E-10	0.0011	0.0011	0.0070	0.0001	0.0003	0.0002	0.0057	0.0000
OTU_254	unclassified Lachnospiraceae	0.929	6.70E-10	0.0071	0.0134	0.0000 *	0.0112	0.0024	0.0040	0.0000	0.0023
OTU_63	unclassified Lachnospiraceae	0.971	6.70E-10	0.0032	0.0064	0.0000 *	0.0060	0.0011	0.0018	0.0000	0.0012
OTU_92	Clostridium XIVa sp.	1	9.70E-10	0.0034 *	0.0022	0.0016	0.0008 *	0.0004	0.0003	0.0002	0.0002
OTU_7	unclassified Lachnospiraceae	1	1.64E-09	0.0189 *	0.0524	0.0580	0.0088 *	0.0037	0.0095	0.0092	0.0014
OTU_5	unclassified Lachnospiraceae	1	2.91E-09	0.1109 *	0.0000	0.0310	0.0071	0.0207	0.0000	0.0065	0.0037
OTU_67	unclassified Lachnospiraceae	1	6.66E-09	0.0087 *	0.0024	0.0018	0.0013	0.0011	0.0003	0.0002	0.0003
OTU_10	Lachnospiraceae bacterium A2	1	8.27E-09	0.0018	0.0012	0.0000	0.0391 *	0.0004	0.0008	0.0000	0.0086
OTU_305	unclassified Lachnospiraceae	0.918	1.26E-08	0.0000	0.0008	0.0008	0.0154 *	0.0000	0.0003	0.0003	0.0022
OTU_139	Marvinbryantia sp.	1	5.22E-08	0.0001 *	0.0012	0.0019	0.0002 *	0.0000	0.0003	0.0004	0.0001
OTU_279	Lachnospiraceae bacterium A2	0.917	7.35E-08	0.0006	0.0005	0.0000	0.0127 *	0.0001	0.0004	0.0000	0.0032
OTU_187	unclassified Lachnospiraceae	1	2.07E-07	0.0004 *	0.0000	0.0000	0.0000	0.0001	0.0000	0.0000	0.0000

Supplemental Table 1

OTU_77	Clostridium XlVa sp.	1	2.07E-07	0.0047 *	0.0022	0.0023	0.0007	0.0008	0.0005	0.0005	0.0002
OTU_170	Anaerotruncus sp.	1	2.73E-07	0.0002	0.0001	0.0003	0.0006 *	0.0001	0.0000	0.0001	0.0001
OTU_86	unclassified Ruminococcaceae	1	3.01E-07	0.0018 *	0.0034	0.0024	0.0012 *	0.0003	0.0004	0.0003	0.0001
OTU_41	unclassified Clostridiales	1	3.11E-07	0.0003	0.0002	0.0001	0.0003	0.0001	0.0001	0.0000	0.0000
OTU_140	Anaerovorax	1	3.11E-07	0.0003 *	0.0008	0.0008	0.0002 *	0.0000	0.0001	0.0002	0.0001
OTU_146	unclassified Ruminococcaceae	1	3.99E-07	0.0000	0.0004	0.0005	0.0012 *	0.0000	0.0001	0.0002	0.0003
OTU_12	Lachnospiraceae bacterium 610	0.95	4.12E-07	0.0360	0.0221	0.0141	0.0413 *	0.0033	0.0062	0.0029	0.0058
OTU_56	Clostridium XlVb sp.	1	5.96E-07	0.0062	0.0043	0.0051	0.0024	0.0007	0.0005	0.0012	0.0003
OTU_96	unclassified Lachnospiraceae	0.893	6.91E-07	0.0002 *	0.0057	0.0064	0.0028	0.0001	0.0017	0.0016	0.0007
OTU_114	Clostridium XlVa sp.	1	1.94E-06	0.0000	0.0001	0.0002	0.0028 *	0.0000	0.0001	0.0001	0.0008
OTU_51	unclassified Lachnospiraceae	1	2.25E-06	0.0068 *	0.0165	0.0144	0.0077 *	0.0011	0.0017	0.0027	0.0011
OTU_6	Oscillibacter sp.	1	3.32E-06	0.0319	0.0437	0.0227 *	0.0257 *	0.0036	0.0033	0.0047	0.0023
OTU_100	Clostridium XlVb sp.	0.895	4.67E-06	0.0019	0.0023	0.0019	0.0006 *	0.0004	0.0004	0.0003	0.0002
OTU_45	unclassified Lachnospiraceae	1	6.63E-06	0.0071	0.0094	0.0000 *	0.0107	0.0024	0.0030	0.0000	0.0031
OTU_147	unclassified Lachnospiraceae	1	1.28E-05	0.0006	0.0006	0.0007	0.0002	0.0001	0.0001	0.0002	0.0000
OTU_129	unclassified Lachnospiraceae	1	1.40E-05	0.0004	0.0001	0.0008	0.0014 *	0.0001	0.0001	0.0006	0.0003
OTU_116	unclassified Lachnospiraceae	1	1.51E-05	0.0035 *	0.0000	0.0000	0.0042 *	0.0010	0.0000	0.0000	0.0011
OTU_123	Alistipes finegoldii	1	1.81E-05	0.0005	0.0002	0.0000	0.0003	0.0002	0.0001	0.0000	0.0001
OTU_57	unclassified Ruminococcaceae	1	2.23E-05	0.0032 *	0.0084	0.0025 *	0.0023 *	0.0007	0.0011	0.0008	0.0006
OTU_142	Pseudomonas mucidolens (T)	1	2.37E-05	0.0002	0.0007	0.0008	0.0001 *	0.0000	0.0001	0.0003	0.0000
OTU_281	unclassified Lachnospiraceae	1	2.59E-05	0.0004	0.0123	0.0096	0.0049	0.0001	0.0040	0.0057	0.0013
OTU_99	Lachnospiraceae	1	2.67E-05	0.0009	0.0016	0.0026 *	0.0013	0.0001	0.0004	0.0003	0.0001
OTU_163	Clostridium IV sp.	1	2.67E-05	0.0000	0.0000	0.0004 *	0.0000	0.0000	0.0000	0.0001	0.0000
OTU_44	Clostridium sp.	1	3.73E-05	0.0166 *	0.0017	0.0056	0.0067	0.0059	0.0009	0.0013	0.0006
OTU_82	Clostridium XlVa sp.	1	3.76E-05	0.0050 *	0.0005	0.0024	0.0017	0.0015	0.0002	0.0005	0.0005
OTU_74	Robinsoniella	1	4.62E-05	0.0005	0.0049	0.0082	0.0057	0.0001	0.0011	0.0036	0.0028
OTU_2	Lachnospiraceae bacterium 14-	1	5.39E-05	0.0817 *	0.0101	0.0940 *	0.0454	0.0168	0.0082	0.0230	0.0162
OTU_59	unclassified Lachnospiraceae	1	5.75E-05	0.0049	0.0058	0.0034 *	0.0024 *	0.0008	0.0006	0.0004	0.0004
OTU_65	Clostridiales bacterium CIEAF 0	0.942	6.50E-05	0.0044	0.0037	0.0006	0.0043	0.0016	0.0013	0.0002	0.0009
OTU_242	unclassified Lachnospiraceae	0.967	8.07E-05	0.0006 *	0.0152	0.0113	0.0054	0.0001	0.0051	0.0063	0.0015
OTU_36	Clostridiales bacterium CIEAF 0	1	9.77E-05	0.0090	0.0138	0.0121	0.0054 *	0.0014	0.0018	0.0031	0.0008
OTU_262	unclassified Lachnospiraceae	0.987	1.13E-04	0.0000	0.0000	0.0082 *	0.0000	0.0000	0.0000	0.0017	0.0000
OTU_22	Clostridium XlVa sp.	1	1.78E-04	0.0199	0.0151	0.0150	0.0106	0.0019	0.0012	0.0016	0.0011
OTU_39	unclassified Lachnospiraceae	1	2.78E-04	0.0136	0.0284	0.0001 *	0.0078	0.0072	0.0077	0.0000	0.0053

Supplemental Table 1

OTU_109	unclassified Ruminococcaceae	1	3.27E-04	0.0003 *	0.0010	0.0004 *	0.0002 *	0.0001	0.0002	0.0001	0.0001
OTU_47	Clostridium XlVa sp.	1	3.27E-04	0.0011	0.0063	0.0101	0.0011	0.0003	0.0018	0.0029	0.0005
OTU_60	Lachnospiracea incertae sedis	1	3.27E-04	0.0038	0.0067	0.0060	0.0012 *	0.0007	0.0021	0.0014	0.0005
OTU_81	unclassified Lachnospiraceae	1	3.41E-04	0.0020 *	0.0060	0.0004 *	0.0004 *	0.0007	0.0015	0.0002	0.0001
OTU_131	Butyricicoccus sp.	1	4.19E-04	0.0002 *	0.0007	0.0008	0.0005	0.0000	0.0002	0.0002	0.0001
OTU_50	unclassified Lachnospiraceae	1	4.19E-04	0.0001	0.0000	0.0270 *	0.0000	0.0001	0.0000	0.0062	0.0000
OTU_173	Elizabethkingia meningoseptica	1	4.71E-04	0.0001 *	0.0003	0.0003	0.0001 *	0.0000	0.0001	0.0001	0.0000
OTU_172	unclassified Lachnospiraceae	1	4.83E-04	0.0004	0.0004	0.0004	0.0000	0.0001	0.0002	0.0002	0.0000
OTU_94	Clostridium IV sp.	1	5.39E-04	0.0070 *	0.0011	0.0005	0.0006	0.0011	0.0003	0.0003	0.0001
OTU_169	Clostridium XlVa sp.	0.934	5.51E-04	0.0005	0.0004	0.0009 *	0.0002	0.0001	0.0000	0.0001	0.0001
OTU_68	segmented filamentous bacteri	1	5.81E-04	0.0000	0.0005	0.0007	0.0002	0.0000	0.0002	0.0004	0.0001
OTU_108	Enterococcus faecalis	1	6.00E-04	0.0002	0.0020	0.0032	0.0005	0.0001	0.0005	0.0012	0.0001
OTU_103	Clostridium fusiformis	0.912	8.46E-04	0.0017	0.0015	0.0012	0.0006	0.0003	0.0002	0.0004	0.0001
OTU_196	Clostridium orbiscindens (T)	0.91	1.11E-03	0.0002 *	0.0000	0.0001	0.0000	0.0000	0.0000	0.0000	0.0000
OTU_75	unclassified Firmicutes	1	1.47E-03	0.0035 *	0.0017	0.0022	0.0018	0.0004	0.0002	0.0003	0.0002
OTU_34	Lachnospiraceae bacterium 615	0.941	1.70E-03	0.0022	0.0038	0.0208	0.0108	0.0007	0.0027	0.0095	0.0024
OTU_159	Clostridium IV sp.	1	2.20E-03	0.0000	0.0002	0.0000	0.0004	0.0000	0.0001	0.0000	0.0001
OTU_231	unclassified Ruminococcaceae	1	3.87E-03	0.0000	0.0000	0.0000	0.0001 *	0.0000	0.0000	0.0000	0.0000
OTU_53	unclassified Lachnospiraceae	1	3.87E-03	0.0000	0.0000	0.0013	0.0162 *	0.0000	0.0000	0.0006	0.0074
OTU_49	Bacteroides caccae	1	4.27E-03	0.0032	0.0020	0.0081 *	0.0005	0.0013	0.0007	0.0022	0.0003
OTU_26	unclassified Ruminococcaceae	1	4.83E-03	0.0072 *	0.0178	0.0166	0.0073 *	0.0006	0.0035	0.0037	0.0010
OTU_193	alpha proteobacterium VUN100	1	5.14E-03	0.0001	0.0001	0.0002	0.0001	0.0000	0.0000	0.0000	0.0000
OTU_31	unclassified Lachnospiraceae	1	5.69E-03	0.0036	0.0000	0.0000	0.0486 *	0.0021	0.0000	0.0000	0.0154
OTU_72	Enterorhabdus sp.	0.97	5.69E-03	0.0018	0.0018	0.0034	0.0008	0.0003	0.0002	0.0011	0.0002
OTU_175	unclassified Lachnospiraceae	1	6.38E-03	0.0000	0.0000	0.0000	0.0008 *	0.0000	0.0000	0.0000	0.0004
OTU_290	unclassified Lachnospiraceae	0.971	6.38E-03	0.0002	0.0004	0.0009 *	0.0002	0.0000	0.0001	0.0002	0.0001
OTU_66	unclassified Lachnospiraceae	1	6.62E-03	0.0068 *	0.0000	0.0041	0.0025	0.0032	0.0000	0.0013	0.0017
OTU_141	unclassified Clostridiales	1	6.98E-03	0.0002	0.0002	0.0003	0.0010 *	0.0001	0.0001	0.0001	0.0002
OTU_162	Oscillibacter sp.	0.98	7.25E-03	0.0002	0.0001	0.0002	0.0006 *	0.0001	0.0000	0.0001	0.0001
OTU_183	Anaerovorax sp.	1	8.27E-03	0.0001	0.0001	0.0005 *	0.0001	0.0001	0.0000	0.0001	0.0000
OTU_4	unclassified Lachnospiraceae	1	9.77E-03	0.0155 *	0.0499	0.0266	0.0258	0.0035	0.0095	0.0095	0.0108
OTU_297	Clostridium fusiformis	0.946	9.77E-03	0.0054	0.0004	0.0003	0.0000	0.0038	0.0002	0.0001	0.0000
OTU_148	Anaerotruncus sp.	1	1.27E-02	0.0007	0.0005	0.0003	0.0003	0.0001	0.0001	0.0001	0.0001
OTU_158	unclassified Lachnospiraceae	1	1.27E-02	0.0003	0.0003	0.0001 *	0.0002	0.0001	0.0001	0.0000	0.0001

Supplemental Table 1

OTU_136	Clostridium XlVa sp.	1	1.33E-02	0.0003	0.0000	0.0000	0.0002	0.0001	0.0000	0.0000	0.0002
OTU_324	unclassified Lachnospiraceae	0.938	1.45E-02	0.0015 *	0.0006	0.0004	0.0003	0.0003	0.0002	0.0002	0.0001
OTU_15	Clostridium cocleatum	1	1.58E-02	0.0067	0.0144	0.0079	0.0029 *	0.0026	0.0029	0.0044	0.0018
OTU_191	unclassified Lachnospiraceae	1	1.68E-02	0.0004 *	0.0000	0.0000	0.0000	0.0001	0.0000	0.0000	0.0000
OTU_337	Oscillibacter sp.	0.942	1.72E-02	0.0013	0.0029	0.0034	0.0023	0.0004	0.0007	0.0005	0.0004
OTU_203	Clostridium XlVa sp.	1	1.75E-02	0.0002 *	0.0001	0.0000	0.0000	0.0000	0.0000	0.0000	0.0000
OTU_25	Barnesiella sp.	1	1.75E-02	0.0240 *	0.0060	0.0134	0.0137	0.0068	0.0013	0.0047	0.0033
OTU_160	Parvibacter caecicola	0.932	1.93E-02	0.0004	0.0001	0.0004	0.0001	0.0001	0.0001	0.0001	0.0000
OTU_157	unclassified Lachnospiraceae	1	1.95E-02	0.0004	0.0004	0.0007	0.0002	0.0001	0.0001	0.0002	0.0001
OTU_69	Eubacterium plexicaudatum	0.971	2.21E-02	0.0000	0.0000	0.0041	0.0038	0.0000	0.0000	0.0022	0.0016
OTU_64	Saccharibacteria genera incerta	1	2.44E-02	0.0012	0.0000	0.0085	0.0058	0.0005	0.0000	0.0062	0.0025
OTU_151	unclassified Lachnospiraceae	1	2.48E-02	0.0005	0.0002	0.0002	0.0004	0.0001	0.0000	0.0001	0.0001
OTU_88	Clostridium XlVa sp.	0.942	2.48E-02	0.0024	0.0006	0.0013	0.0037 *	0.0009	0.0003	0.0006	0.0009
OTU_23	Alistipes sp.	1	2.63E-02	0.0025	0.0047	0.0097 *	0.0054	0.0008	0.0008	0.0023	0.0008
OTU_1	Lachnospiraceae bacterium 609	1	2.67E-02	0.0355 *	0.1086	0.0436 *	0.0247 *	0.0152	0.0190	0.0180	0.0146
OTU_78	Clostridium XlVa	1	2.68E-02	0.0039	0.0023	0.0036	0.0020	0.0009	0.0002	0.0005	0.0004
OTU_83	Clostridiales bacterium CIEAF 0	0.951	2.72E-02	0.0035	0.0056	0.0062	0.0043	0.0004	0.0013	0.0009	0.0008
OTU_130	unclassified Lachnospiraceae	1	2.77E-02	0.0004	0.0005	0.0009	0.0002	0.0001	0.0002	0.0003	0.0000
OTU_112	unclassified Lachnospiraceae	1	2.87E-02	0.0000	0.0000	0.0006 *	0.0001	0.0000	0.0000	0.0002	0.0000
OTU_61	Shigella boydii	1	3.26E-02	0.0002 *	0.0129	0.0029	0.0037	0.0001	0.0050	0.0012	0.0024
OTU_93	unclassified Ruminococcaceae	0.984	3.73E-02	0.0034	0.0024	0.0055 *	0.0023	0.0007	0.0006	0.0010	0.0003
OTU_9	Clostridiales bacterium CIEAF 0	0.971	3.73E-02	0.0083 *	0.0628	0.0046 *	0.0000 *	0.0081	0.0238	0.0046	0.0000
OTU_227	unclassified Ruminococcaceae	0.975	4.43E-02	0.0020	0.0019	0.0009	0.0029	0.0003	0.0004	0.0003	0.0008
OTU_101	unclassified Lachnospiraceae	0.946	4.74E-02	0.0103 *	0.0000	0.0000	0.0000	0.0027	0.0000	0.0000	0.0000

Supplemental Table 2

name	rank	R_FDR_p	LFD	WD	RS2	RS4	SEM LFD	SEM WD	SEM RS2	SEM RS4
Bacteroidetes	phylum	1,85E-03	0,1023	0,0526	0,0827	0,1384 *	2,02E-02	7,14E-03	1,73E-02	2,34E-02
Actinobacteria	phylum	5,35E-03	0,0023	0,0019	0,0039	0,0010	3,98E-04	1,74E-04	1,21E-03	2,44E-04
Proteobacteria	phylum	2,12E-02	0,0013 *	0,0148	0,0055	0,0044	3,68E-04	5,28E-03	1,46E-03	2,46E-03
TM7	phylum	3,58E-02	0,0012	0,0000	0,0085	0,0058	5,03E-04	0,00E+00	6,18E-03	2,49E-03
Pseudomonadales	order	1,28E-04	0,0002	0,0007	0,0009	0,0002	5,03E-05	1,18E-04	3,26E-04	3,82E-05
Bacteroidales	order	1,85E-03	0,1020	0,0520	0,0820	0,1382 *	2,01E-02	7,17E-03	1,73E-02	2,34E-02
Coriobacteriales	order	5,35E-03	0,0023	0,0019	0,0038	0,0010	3,97E-04	1,72E-04	1,18E-03	2,44E-04
Caulobacterales	order	8,83E-03	0,0001	0,0001	0,0002	0,0001	4,46E-05	4,49E-05	4,66E-05	1,86E-05
Flavobacteriales	order	2,12E-02	0,0002	0,0004	0,0005	0,0002	5,65E-05	1,04E-04	1,74E-04	3,17E-05
Erysipelotrichales	order	2,38E-02	0,0069	0,0145	0,0082	0,0030 *	2,61E-03	2,96E-03	4,39E-03	1,83E-03
Enterobacteriales	order	2,38E-02	0,0004 *	0,0134	0,0038	0,0039	1,47E-04	5,21E-03	1,26E-03	2,49E-03
Bacteroidia	class	1,85E-03	0,1020	0,0520	0,0820	0,1382 *	2,01E-02	7,17E-03	1,73E-02	2,34E-02
Actinobacteria	class	5,35E-03	0,0023	0,0019	0,0039	0,0010	3,98E-04	1,74E-04	1,21E-03	2,44E-04
Gammaproteobacteria	class	1,14E-02	0,0008 *	0,0143	0,0048	0,0041	1,89E-04	5,23E-03	1,34E-03	2,49E-03
Flavobacteria	class	2,12E-02	0,0002	0,0004	0,0005	0,0002	5,65E-05	1,04E-04	1,74E-04	3,17E-05
Erysipelotrichia	class	2,38E-02	0,0069	0,0145	0,0082	0,0030 *	2,61E-03	2,96E-03	4,39E-03	1,83E-03
Ruminococcaceae	family	3,43E-09	0,0828 *	0,1203	0,0969	0,0668 *	5,28E-03	8,36E-03	1,06E-02	2,85E-03
Clostridiales_Incertae Sedis XIII	family	5,97E-07	0,0003 *	0,0007	0,0009	0,0002 *	3,83E-05	8,60E-05	1,55E-04	7,40E-05
Pseudomonadaceae	family	7,61E-05	0,0002	0,0007	0,0009	0,0002 *	4,50E-05	1,18E-04	3,00E-04	4,00E-05
Enterococcaceae	family	1,85E-03	0,0002	0,0020	0,0032	0,0005	7,17E-05	5,35E-04	1,19E-03	1,32E-04
Bacteroidaceae	family	3,91E-03	0,0110	0,0075	0,0136	0,0402 *	2,20E-03	1,21E-03	2,91E-03	9,21E-03
Coriobacteriaceae	family	5,35E-03	0,0023	0,0019	0,0038	0,0010	3,97E-04	1,72E-04	1,18E-03	2,44E-04
Caulobacteraceae	family	8,83E-03	0,0001	0,0001	0,0002	0,0001	4,46E-05	4,49E-05	4,66E-05	1,86E-05
Flavobacteriaceae	family	2,12E-02	0,0002	0,0004	0,0005	0,0002	5,65E-05	1,04E-04	1,74E-04	3,17E-05
Erysipelotrichaceae	family	2,38E-02	0,0069	0,0145	0,0082	0,0030 *	2,61E-03	2,96E-03	4,39E-03	1,83E-03
Enterobacteriaceae	family	2,38E-02	0,0004 *	0,0134	0,0038	0,0039	1,47E-04	5,21E-03	1,26E-03	2,49E-03
Rikenellaceae	family	3,72E-02	0,0030	0,0049	0,0097 *	0,0057	7,65E-04	8,17E-04	2,29E-03	8,28E-04
Clostridium IV	genus	2,44E-15	0,0070 *	0,0037	0,0039	0,0007 *	1,12E-03	8,08E-04	5,85E-04	1,14E-04
Clostridium XlVb	genus	9,46E-08	0,0025	0,0026	0,0023	0,0007 *	3,65E-04	4,61E-04	3,33E-04	1,60E-04
Blautia	genus	9,46E-08	0,0014 *	0,0008	0,0008	0,0002	2,13E-04	1,74E-04	1,77E-04	7,65E-05

Supplemental Table 2

Anaerovorax	genus	4,92E-07	0,0002 *	0,0007	0,0008	0,0002 *	3,96E-05	8,19E-05	1,47E-04	7,49E-05
Oscillibacter	genus	8,96E-06	0,0282 *	0,0433	0,0257 *	0,0270 *	2,80E-03	2,74E-03	3,87E-03	2,20E-03
Pseudomonas	genus	7,61E-05	0,0002	0,0007	0,0009	0,0002 *	4,50E-05	1,18E-04	2,89E-04	4,00E-05
Elizabethkingia	genus	1,65E-03	0,0001 *	0,0003	0,0003	0,0001 *	2,91E-05	5,60E-05	5,63E-05	2,29E-05
Flavonifractor	genus	1,85E-03	0,0007 *	0,0016	0,0007 *	0,0004 *	1,05E-04	2,40E-04	3,39E-04	1,43E-04
Enterococcus	genus	1,85E-03	0,0002	0,0020	0,0032	0,0005	7,17E-05	5,35E-04	1,18E-03	1,33E-04
Bacteroides	genus	3,91E-03	0,0110	0,0075	0,0136	0,0402 *	2,20E-03	1,21E-03	2,91E-03	9,21E-03
Brevundimonas	genus	8,83E-03	0,0001	0,0001	0,0002	0,0001	4,46E-05	4,49E-05	4,66E-05	1,86E-05
Enterorhabdus	genus	1,00E-02	0,0018	0,0018	0,0034	0,0008	3,17E-04	1,97E-04	1,08E-03	2,39E-04
Butyricicoccus	genus	1,44E-02	0,0002	0,0004	0,0005	0,0003	5,65E-05	9,28E-05	9,86E-05	9,00E-05
Clostridium XVIII	genus	2,38E-02	0,0067	0,0144	0,0079	0,0029 *	2,61E-03	2,94E-03	4,45E-03	1,84E-03
TM7_genera_incertae_sedis	genus	3,58E-02	0,0012	0,0000	0,0085	0,0058	5,03E-04	0,00E+00	6,18E-03	2,49E-03
Alistipes	genus	3,72E-02	0,0030	0,0049	0,0097 *	0,0057	7,65E-04	8,17E-04	2,29E-03	8,28E-04
Escherichia/Shigella	genus	4,88E-02	0,0002 *	0,0129	0,0029	0,0037	1,24E-04	5,04E-03	1,18E-03	2,42E-03
Coriobacteridae	subclass	5,35E-03	0,0023	0,0019	0,0038	0,0010	3,97E-04	1,72E-04	1,18E-03	2,44E-04
Coriobacterineae	suborder	5,35E-03	0,0023	0,0019	0,0038	0,0010	3,97E-04	1,72E-04	1,18E-03	2,44E-04
unclassified_Bacteria		4,03E-11	0,0471 *	0,1410	0,0666 *	0,0493 *	1,09E-02	9,56E-03	1,65E-02	1,53E-02
unclassified_Ruminococcaceae		1,41E-09	0,0448 *	0,0697	0,0621	0,0368 *	3,39E-03	6,00E-03	7,68E-03	7,62E-04
unclassified_Clostridiales		3,91E-03	0,1529 *	0,0886	0,0824	0,1215	1,88E-02	7,34E-03	1,17E-02	1,09E-02
unclassified_Flavobacteriaceae		8,23E-03	0,0000	0,0000	0,0000	0,0000	0,00E+00	1,75E-05	2,21E-05	1,63E-05
unclassified_Coriobacteriaceae		3,58E-02	0,0005	0,0002	0,0004	0,0002	9,95E-05	5,79E-05	1,22E-04	4,33E-05
unclassified_Clostridia		4,90E-02	0,0014	0,0019	0,0016	0,0025	1,71E-04	8,74E-05	2,33E-04	3,43E-04

Supplemental Table 3

MEAN	*p<0.05 Microbial status #p<0.05 Diet 2w AOV	CVZ-LFD	CVZ-WD	CVZ-RS2	CVZ-RS4	GF-LFD	GF-WD	GF-RS2	GF-RS4
CA	CA *	158.66	227.90	193.02	123.88	0.07	0.09	0.11	0.10
DCA	DCA *#	677.81	763.63	560.53	335.46	1.06	1.04	1.27	1.36
LCA	LCA *	4.97	7.73	7.43	4.15	0.01	0.01	0.01	0.01
ACA	ACA *	38.53	39.82	43.49	34.16	0.17	0.13	0.14	0.10
CDCA	CDCA *	1.66	3.36	1.54	2.95	0.00	0.00	0.00	0.00
DHCA	DHCA *#	0.02	0.03	0.05	0.05	0.00	0.01	0.01	0.01
12-KLCA	12-KLCA *	7.79	6.64	5.57	4.20	0.02	0.04	0.01	0.01
DHLCA	DHLCA *	0.23	0.28	0.25	0.14	0.00	0.00	0.00	0.00
7-KDCA	7-KDCA *	13.27	16.67	11.76	5.59	0.18	0.38	0.41	0.33
7-KLCA	7-KLCA *	0.69	0.88	0.50	0.58	0.00	0.00	0.00	0.00
AILCA	AILCA *	0.15	0.12	0.15	0.13	0.00	0.00	0.00	0.00
ApoCA	ApoCA *	0.21	0.19	0.21	0.14	0.00	0.00	0.00	0.00
HDCA	HDCA *	3.46	3.54	2.82	1.24	0.20	0.18	0.15	0.16
MuroCA	MuroCA *	1.32	1.29	0.77	0.90	0.00	0.00	0.00	0.00
UDCA	UDCA *	14.56	17.24	11.15	10.44	0.01	0.01	0.02	0.01
DioLCA	DioLCA *#	0.63	0.42	0.66	0.31	0.00	0.00	0.00	0.00
ILCA	ILCA *	0.85	1.27	0.91	0.79	0.00	0.00	0.00	0.00
IDCA	IDCA	0.02	0.00	0.00	0.00	0.00	0.00	0.00	0.00
12-KCDCA	12-KCDCA *	2.99	4.05	2.69	2.12	0.00	0.01	0.01	0.00
23-NDCA	23-NDCA *#	0.01	0.01	0.01	0.01	0.00	0.01	0.00	0.00
3-OCA	3-OCA *	0.72	0.81	0.74	0.60	0.00	0.00	0.00	0.00
6,7-DiKLCA	6,7-DiKLCA *	0.06	0.07	0.08	0.07	0.00	0.02	0.04	0.01
aMCA	aMCA *#	399.81	597.98	293.38	204.99	7.98	7.59	7.83	8.23
bMCA	bMCA *	139.32	157.08	119.51	94.76	0.28	0.44	0.80	0.44
HCA	HCA *	4.55	7.44	5.25	3.75	0.01	0.03	0.03	0.02
wMCA	wMCA *#	1562.13	1986.59	901.84	612.47	0.96	0.90	0.91	0.93
GCA	GCA *#	0.12	0.16	0.12	0.12	0.03	0.08	0.04	0.02
CDCA	CDCA *	0.09	0.06	0.14	0.10	0.00	0.00	0.00	0.00
GUDCA	GUDCA *	0.01	0.02	0.01	0.01	0.00	0.01	0.01	0.01
GLCA	GLCA *	0.01	0.01	0.01	0.01	0.00	0.00	0.00	0.00
GHCA	GHCA *	0.01	0.01	0.00	0.01	0.00	0.00	0.00	0.00
TDCA	TDCA *	33.70	53.10	30.04	20.73	0.00	0.01	0.00	0.00
TCDCA	TCDCA *#	1.15	9.63	1.31	3.34	2.38	8.31	5.35	3.04
TCA	TCA *#	73.07	179.98	93.79	73.33	92.19	291.49	196.35	92.86
TDHCA	TDHCA *#	0.01	0.04	0.04	0.02	0.11	0.05	0.05	0.05
TLCA	TLCA *#	1.51	2.13	1.78	0.67	0.01	0.01	0.01	0.01
THDC+TUDC	THDC + TUDC #	17.79	73.37	12.82	24.42	14.51	36.99	36.12	18.83
THC	THC *#	4.14	17.47	5.20	4.37	0.48	0.57	0.65	0.59
TaMCA	TaMCA *#	154.53	627.96	183.08	200.79	74.95	233.98	126.78	94.17
TbMCA	TbMCA *#	538.68	1866.80	396.55	546.74	1160.72	2067.81	1742.24	1357.45
TwMCA	TwMCA *#	235.79	562.72	76.39	86.85	0.37	0.29	0.30	0.30
UCA	UCA *	0.27	0.42	0.44	0.32	0.21	0.00	0.04	0.06
GwMCA	GwMCA *	0.22	0.22	0.20	0.20	0.00	0.00	0.00	0.00
GaMCA	GaMCA *	0.06	0.08	0.07	0.07	0.01	0.04	0.02	0.02
GbMCA	GbMCA #	0.13	0.21	0.20	0.19	0.15	0.30	0.26	0.21
GalloCA	GalloCA *#	0.06	0.11	0.11	0.13	0.04	0.07	0.05	0.04

Supplemental Table 3

SEM	CVZ-LFD	CVZ-WD	CVZ-RS2	CVZ-RS4	GF-LFD	GF-WD	GF-RS2	GF-RS4
CA	25.06	73.06	62.21	17.73	0.03	0.02	0.03	0.02
DCA	106.88	178.27	91.65	47.27	0.05	0.07	0.07	0.20
LCA	0.75	2.34	0.66	1.17	0.00	0.00	0.00	0.00
ACA	6.88	5.90	2.54	4.11	0.03	0.01	0.01	0.01
CDCA	0.57	0.48	0.25	1.46	0.00	0.00	0.00	0.00
DHCA	0.00	0.01	0.01	0.01	0.00	0.00	0.00	0.00
12-KLCA	1.76	2.02	0.83	0.81	0.00	0.01	0.00	0.00
DHLCA	0.03	0.09	0.05	0.04	0.00	0.00	0.00	0.00
7-KDCA	4.91	2.90	1.78	0.74	0.02	0.05	0.04	0.02
7-KLCA	0.21	0.22	0.08	0.16	0.00	0.00	0.00	0.00
AILCA	0.02	0.04	0.03	0.01	0.00	0.00	0.00	0.00
ApoCA	0.04	0.06	0.03	0.02	0.00	0.00	0.00	0.00
HDCA	0.98	1.12	0.59	0.23	0.04	0.03	0.01	0.04
MuroCA	0.30	0.41	0.16	0.16	0.00	0.00	0.00	0.00
UDCA	3.77	4.75	1.69	1.98	0.00	0.00	0.00	0.00
DioLCA	0.09	0.10	0.11	0.04	0.00	0.00	0.00	0.00
ILCA	0.13	0.46	0.17	0.23	0.00	0.00	0.00	0.00
IDCA	0.01	0.00	0.00	0.00	0.00	0.00	0.00	0.00
12-KCDCA	0.76	1.11	0.44	0.36	0.00	0.00	0.00	0.00
23-NDCA	0.00	0.00	0.00	0.00	0.00	0.00	0.00	0.00
3-OCA	0.18	0.22	0.22	0.15	0.00	0.00	0.00	0.00
6,7-DiKLCA	0.01	0.01	0.01	0.01	0.00	0.00	0.00	0.00
aMCA	79.17	148.01	34.90	58.28	0.15	0.18	0.22	0.08
bMCA	40.53	41.76	24.68	20.20	0.03	0.02	0.04	0.04
HCA	0.93	2.28	0.27	0.47	0.00	0.00	0.00	0.00
wMCA	366.69	553.28	157.08	79.69	0.02	0.01	0.01	0.02
GCA	0.02	0.04	0.03	0.03	0.01	0.00	0.01	0.01
CDCA	0.01	0.01	0.02	0.02	0.00	0.00	0.00	0.00
GUDCA	0.00	0.00	0.00	0.00	0.00	0.00	0.00	0.00
GLCA	0.00	0.00	0.00	0.00	0.00	0.00	0.00	0.00
GHCA	0.00	0.00	0.00	0.00	0.00	0.00	0.00	0.00
TDCA	10.73	7.23	4.25	6.39	0.00	0.00	0.00	0.00
TCDCA	0.25	1.29	0.16	0.74	0.63	1.28	0.96	0.82
TCA	14.56	38.41	15.69	24.62	19.48	26.25	28.84	12.81
TDHCA	0.00	0.01	0.01	0.01	0.01	0.00	0.00	0.01
TLCA	0.37	0.65	0.30	0.07	0.00	0.00	0.00	0.00
THDC+TUDC	3.96	26.17	0.70	6.31	2.84	5.38	4.28	4.36
THC	0.79	4.06	0.83	0.63	0.14	0.06	0.06	0.06
TaMCA	45.64	189.04	37.28	36.78	24.79	31.45	19.24	27.17
TbMCA	94.64	638.11	91.39	115.17	379.14	247.45	179.87	271.46
TwMCA	49.10	154.90	13.51	12.74	0.15	0.05	0.05	0.08
UCA	0.06	0.10	0.13	0.07	0.02	0.00	0.01	0.01
GwMCA	0.05	0.05	0.06	0.05	0.00	0.00	0.00	0.00
GaMCA	0.02	0.02	0.01	0.01	0.00	0.00	0.00	0.00
GbMCA	0.05	0.06	0.04	0.01	0.05	0.03	0.03	0.05
GalloCA	0.01	0.02	0.03	0.02	0.00	0.00	0.00	0.00

Supplemental Table 3

2w AOV	p.value Microbial status		p.value Diet		p.value Interaction	
CA	7.08E-007	*	1.09E-001		1.26E-001	
DCA	7.20E-013	*	2.83E-002	#	2.81E-002	*
LCA	2.07E-010	*	3.10E-001		3.36E-001	
ACA	6.27E-021	*	6.33E-001		6.64E-001	
CDCA	9.38E-006	*	4.24E-001		4.10E-001	
DHCA	1.69E-005	*	2.74E-002	#	1.47E-001	
12-KLCA	2.01E-010	*	3.53E-001		4.15E-001	
DHLCA	4.71E-009	*	3.84E-001		4.07E-001	
7-KDCA	3.72E-008	*	2.80E-001		3.58E-001	
7-KLCA	1.24E-009	*	4.71E-001		4.77E-001	
AILCA	9.37E-012	*	5.52E-001		5.49E-001	
ApoCA	1.39E-012	*	5.45E-001		5.83E-001	
HDCA	8.04E-008	*	1.57E-001		2.06E-001	
MuroCA	3.96E-010	*	4.04E-001		4.12E-001	
UDCA	2.03E-010	*	4.43E-001		4.69E-001	
DioLCA	2.04E-013	*	2.64E-002	#	2.64E-002	*
ILCA	9.03E-009	*	6.19E-001		6.29E-001	
IDCA	2.43E-001		3.07E-001		2.69E-001	
12-KCDCA	1.01E-010	*	3.02E-001		3.29E-001	
23-NDCA	2.47E-005	*	4.58E-003	#	1.78E-001	
3-OCA	1.97E-007	*	2.97E-001		2.91E-001	
6,7-DiKLCA	4.36E-008	*	1.15E-001		5.07E-001	
aMCA	1.98E-010	*	2.09E-002	#	3.00E-002	*
bMCA	4.99E-010	*	5.81E-001		6.05E-001	
HCA	2.29E-010	*	1.13E-001		1.15E-001	
wMCA	2.94E-009	*	2.25E-002	#	2.25E-002	*
GCA	1.39E-004	*	2.42E-002	#	8.30E-001	
CDCA	7.85E-014	*	2.51E-001		2.65E-001	
GUDCA	3.71E-002	*	1.37E-001		1.40E-002	*
GLCA	1.41E-016	*	8.17E-001		8.31E-001	
GHCA	2.33E-004	*	9.08E-001		5.37E-001	
TDCA	1.45E-010	*	4.59E-001		4.80E-001	
TCDCA	1.28E-004	*	1.19E-006	#	2.65E-001	
TCA	2.80E-004	*	4.59E-008	#	3.24E-002	*
TDHCA	9.40E-009	*	1.62E-002	#	2.07E-006	*
TLCA	2.89E-009	*	4.75E-002	#	5.00E-002	
THDC+TUDC	6.22E-001		1.09E-003	#	4.06E-002	*
THC	7.75E-008	*	3.49E-005	#	4.89E-005	*
TaMCA	1.18E-002	*	9.77E-005	#	5.62E-002	
TbMCA	7.10E-004	*	1.41E-003	#	2.84E-001	
TwMCA	7.33E-007	*	2.03E-004	#	2.03E-004	*
UCA	3.32E-007	*	8.32E-001		4.75E-002	*
GwMCA	3.18E-011	*	9.74E-001		9.76E-001	
GaMCA	6.91E-007	*	6.48E-002		8.23E-001	
GbMCA	6.65E-002		3.90E-002	#	8.64E-001	
GalloCA	1.50E-004	*	3.68E-002	#	2.14E-001	

Supplemental Table 3

1w AOV CVZ	p.value 1w AOV		p.value WD-LFD		p.value WD-RS2		p.value WD-RS4	
CA	1.30E-001		2.92E-001		9.36E-001		9.03E-002	
DCA	3.43E-002	*	9.19E-001	*	4.88E-001		1.63E-002	*
LCA	3.25E-001		3.95E-001		8.28E-001		2.04E-001	
ACA	6.44E-001		9.96E-001		9.22E-001		7.77E-001	
CDCA	4.33E-001		7.30E-001		4.76E-001		9.95E-001	
DHCA	1.03E-001		1.90E-001		8.85E-001		5.39E-001	
12-KLCA	3.67E-001		9.00E-001		9.16E-001		5.10E-001	
DHLCA	4.03E-001		5.94E-001		9.80E-001		2.97E-001	
7-KDCA	3.19E-001		8.91E-001		9.92E-001		5.05E-001	
7-KLCA	4.72E-001		7.94E-001		3.21E-001		4.86E-001	
AILCA	5.62E-001		9.98E-001		6.78E-001		9.43E-001	
ApoCA	5.60E-001		9.69E-001		9.70E-001		7.01E-001	
HDCA	1.79E-001		1.00E+000		8.65E-001		1.30E-001	
MuroCA	4.08E-001		1.00E+000		4.19E-001		6.33E-001	
UDCA	4.46E-001		8.91E-001		4.32E-001		3.45E-001	
DioLCA	3.73E-002	*	9.62E-001	*	1.78E-001		4.44E-001	
ILCA	6.20E-001		5.87E-001		6.76E-001		4.86E-001	
IDCA	2.98E-001		2.48E-001		9.98E-001		1.00E+000	
12-KCDCA	3.17E-001		6.13E-001		4.24E-001		1.73E-001	
23-NDCA	6.36E-002		9.49E-001		1.71E-001		2.42E-001	
3-OCA	3.13E-001		5.16E-001		9.90E-001		2.44E-001	
6,7-DiKLCA	7.74E-001		9.55E-001		7.91E-001		9.93E-001	
aMCA	2.86E-002	*	3.00E-001	*	6.27E-002		1.29E-002	*
bMCA	5.89E-001		9.64E-001		7.64E-001		4.18E-001	
HCA	1.33E-001		2.98E-001		6.84E-001		6.19E-002	
wMCA	3.01E-002	*	7.28E-001	*	9.29E-002		1.87E-002	*
GCA	2.42E-001		2.21E-001		6.16E-001		1.81E-001	
CDCA	2.69E-001		3.47E-001		4.09E-001		1.56E-001	
GUDCA	1.41E-001		9.85E-001		9.52E-002		9.66E-001	
GLCA	8.20E-001		9.69E-001		8.97E-001		9.77E-001	
GHCA	7.37E-001		9.42E-001		8.45E-001		9.98E-001	
TDCA	4.75E-001		9.75E-001		8.44E-001		3.18E-001	
TCDCA	1.50E-003	*	1.77E-003	*	1.93E-003	*	6.70E-003	*
TCA	1.23E-002	*	1.68E-002	*	1.97E-002	*	1.70E-002	*
TDHCA	1.42E-001		5.93E-002		5.18E-001		3.39E-001	
TLCA	5.92E-002		6.00E-001		8.84E-001		2.67E-002	
THDC+TUDC	1.44E-002	*	2.58E-002	*	1.48E-002	*	2.60E-002	*
THC	1.68E-004	*	5.17E-004	*	1.25E-003	*	2.02E-004	*
TaMCA	4.48E-003	*	8.07E-003	*	1.31E-002	*	4.89E-003	*
TbMCA	9.80E-003	*	2.53E-002	*	1.24E-002	*	1.14E-002	*
TwMCA	6.57E-004	*	2.65E-002	*	8.26E-004	*	7.23E-004	*
UCA	5.66E-001		5.48E-001		9.99E-001		8.02E-001	
GwMCA	9.74E-001		9.99E-001		9.88E-001		9.83E-001	
GaMCA	3.99E-001		5.56E-001		9.05E-001		2.54E-001	
GbMCA	5.76E-001		4.88E-001		1.00E+000		8.00E-001	
GalloCA	1.76E-001		1.67E-001		1.00E+000		1.00E+000	

Supplemental Table 3

1w AOV GF	p.value 1w AOV		p.value WD-LFD		p.value WD-RS2		p.value WD-RS4	
CA	6.11E-001		8.78E-001		9.04E-001		8.77E-001	
DCA	1.99E-001		9.97E-001		9.00E-001		1.32E-001	
LCA	6.15E-002		1.10E-001		9.56E-001		9.64E-001	
ACA	8.49E-002		2.99E-001		9.82E-001		5.06E-001	
CDCA	6.95E-001		5.64E-001		9.77E-001		7.63E-001	
DHCA	1.06E-005	*	7.82E-003	*	2.70E-001		1.15E-002	*
12-KLCA	1.41E-002	*	2.60E-002	*	1.54E-002	*	2.66E-002	*
DHLCA	NaN		NaN		NaN		NaN	
7-KDCA	2.90E-004	*	8.64E-004	*	8.77E-001		3.51E-001	
7-KLCA	NaN		NaN		NaN		NaN	
AlLCA	NaN		NaN		NaN		NaN	
ApoCA	NaN		NaN		NaN		NaN	
HDCA	8.27E-001		9.03E-001		9.99E-001		9.67E-001	
MuroCA	NaN		NaN		NaN		NaN	
UDCA	1.89E-003	*	9.99E-001		4.58E-003	*	8.96E-001	
DioLCA	NaN		NaN		NaN		NaN	
ILCA	NaN		NaN		NaN		NaN	
IDCA	NaN		NaN		NaN		NaN	
12-KCDCA	5.72E-004	*	1.50E-002	*	7.06E-001		1.93E-002	*
23-NDCA	2.18E-002	*	3.94E-002	*	3.49E-002	*	1.91E-002	*
3-OCA	NaN		NaN		NaN		NaN	
6,7-DiKLCA	1.25E-010	*	3.52E-005	*	3.78E-006	*	3.91E-002	*
aMCA	2.35E-002	*	2.63E-001		6.23E-001		8.14E-003	*
bMCA	1.31E-010	*	4.67E-003	*	1.54E-008	*	1.00E+000	
HCA	1.10E-003	*	1.51E-003	*	9.99E-001		4.50E-001	
wMCA	8.58E-002		4.02E-002		8.58E-001		4.74E-001	
GCA	8.42E-006	*	3.34E-005	*	4.11E-003	*	6.47E-006	*
CDCA	NaN		NaN		NaN		NaN	
GUDCA	7.21E-004	*	5.95E-004	*	9.73E-001		1.61E-001	
GLCA	NaN		NaN		NaN		NaN	
GHCA	4.29E-001		1.00E+000		3.86E-001		1.00E+000	
TDCA	1.89E-001		1.34E-001		9.61E-001		3.74E-001	
TCDCA	6.14E-004	*	3.51E-004	*	9.08E-002		2.01E-003	*
TCA	1.85E-006	*	2.61E-006	*	2.03E-002	*	4.07E-006	*
TDHCA	1.89E-006	*	4.12E-006	*	1.00E+000		1.00E+000	
TLCA	3.12E-007	*	1.68E-004	*	2.00E-001		6.12E-004	*
THDC+TUDC	9.47E-004	*	2.47E-003	*	9.98E-001		1.89E-002	*
THC	4.72E-001		8.16E-001		8.50E-001		8.23E-001	
TaMCA	7.77E-004	*	4.09E-004	*	1.69E-002	*	2.48E-003	*
TbMCA	1.21E-001		7.06E-002		7.43E-001		2.12E-001	
TwMCA	9.07E-001		8.38E-001		9.98E-001		9.99E-001	
UCA	2.89E-011	*	7.11E-015	*	3.21E-001		1.60E-001	
GwMCA	NaN		NaN		NaN		NaN	
GaMCA	5.50E-004	*	2.26E-004	*	2.12E-002	*	2.70E-003	*
GbMCA	4.29E-002	*	1.91E-002	*	7.58E-001		2.54E-001	
GalloCA	1.00E-005	*	4.64E-005	*	4.30E-003	*	1.34E-005	*

Supplemental Table 3

**Pearson r**	CD11c (SAT)	F4/80 (SAT)	insulin (pM)	HOMA-IR
Isolithocholic acid	-0.0352546945	0.014742746	-0.1796823442	-0.2054184228
Taurochenodeoxycholic acid	0.3550248742	0.3751581609	0.5346249342	0.5547686815
Murocholic acid	-0.0070430548	0.0471135415	-0.1382373273	-0.1715968102
Taurolithocholic acid	0.0812138692	0.1023225933	-0.0739841834	-0.1026603356
Chenodeoxycholic acid	0.267929107	0.2461850792	-0.1450288892	-0.1513807476
12-Ketochenodeoxycholic acid	0.0756820887	0.1205104291	-0.1208036616	-0.1517657042
Hyodeoxycholic acid	-0.0115417056	0.0305692274	-0.1746860147	-0.1865325272
Taurohyocholic acid	0.2159255594	0.2120873183	-0.0175491292	-0.0451331511
Hyocholic acid	0.0611284152	0.1148756668	-0.152200222	-0.1752488315
Lithocholic acid	0.045684617	0.0849980712	-0.1502563655	-0.1771179885
12-Ketolithocholic acid	-0.0567168072	0.0141955679	-0.2116084397	-0.2368295044
7-Ketodeoxycholic acid	-0.0424398035	0.0041016294	-0.2114225477	-0.2244059294
Ursodeoxycholic acid	0.0774641186	0.1000497714	-0.1422722936	-0.1744169891
Taurohyodeoxycholic and Tauroursodeoxycholic acid	0.2806141973	0.2735173702	0.1688374728	0.1670140177
Taurodeoxycholic acid	0.1267015487	0.1752401888	-0.1436080188	-0.1601172984
Allocholic acid	0.1536984593	0.1961731017	-0.169279024	-0.1943936795
Taurocholic acid	0.3594596684	0.3717797697	0.5365024805	0.5525179505
β-muricholic acid	0.1071741655	0.1248981357	-0.0841944143	-0.1172606573
Tauro-α-muricholic acid	0.2905148864	0.2581671774	0.2152154595	0.1972515881
Cholic acid	0.1749079973	0.1561232358	-0.0268603656	-0.067033872
Tauro-ω-muricholic acid	0.1751672477	0.1600849777	-0.0446925797	-0.0624160729
α-muricholic acid	0.1850809455	0.1993665099	-0.0457266793	-0.0834447891
Tauro-β-muricholic acid	0.1243511736	0.1125323996	0.3187763393	0.3227938116
Deoxycholic acid	0.0873883069	0.1330688298	-0.1334968358	-0.1637497097
Ω-muricholic acid	0.0764191896	0.1214735582	-0.1147332191	-0.1408189237

Legend
$p<0.05$
$q<0.05$

Supplemental Table 3

**P-values**	CD11c (SAT)	F4/80 (SAT)	insulin (pM)	HOMA-IR
Isolithocholic acid	0.7855982262	0.9094532571	0.1622843503	0.1092260272
Taurochenodeoxycholic acid	0.0057950797	0.0034143497	0.000012903	5.127413E-006
Murocholic acid	0.9566729555	0.716138227	0.28395161	0.1823437951
Taurolithocholic acid	0.5338054914	0.4326325308	0.5709463601	0.4311070553
Chenodeoxycholic acid	0.0384786107	0.0579437809	0.2688931797	0.2482574836
12-Ketochenodeoxycholic acid	0.55879435	0.3508224348	0.3496428811	0.2389878738
Hyodeoxycholic acid	0.9296528397	0.8150869684	0.1781428253	0.1500390004
Taurohyocholic acid	0.0975237255	0.1037734194	0.894125653	0.7320346394
Hyocholic acid	0.6426674837	0.3821165471	0.2456760358	0.1804707164
Lithocholic acid	0.7288854363	0.5184656326	0.251829408	0.1757935055
12-Ketolithocholic acid	0.6668817178	0.9142727863	0.1045744265	0.0684704892
7-Ketodeoxycholic acid	0.7474776271	0.9751875061	0.1048866479	0.0847557255
Ursodeoxycholic acid	0.5529199001	0.4429769971	0.2740560893	0.1788225575
Taurohyodeoxycholic and Tauroursodeoxycholic acid	0.0298749259	0.0344645453	0.1971968883	0.202149612
Taurodeoxycholic acid	0.3305397356	0.1767485322	0.2695299228	0.2177041333
Allocholic acid	0.2329884502	0.1264818402	0.1884112214	0.1300282676
Taurocholic acid	0.0047913376	0.0034463402	9.936188E-006	4.713898E-006
β-muricholic acid	0.4070432652	0.3334295709	0.5152966243	0.3640590994
Tauro-α-muricholic acid	0.0231299402	0.0445550548	0.0929827109	0.1243684118
Cholic acid	0.1851750022	0.23357231	0.8385727323	0.6108276673
Tauro-ω-muricholic acid	0.1769315969	0.217798237	0.7301541881	0.6298533984
α-muricholic acid	0.1532936182	0.1234632876	0.7263900883	0.5225898111
Tauro-β-muricholic acid	0.3396615369	0.387878673	0.0115625518	0.0105018255
Deoxycholic acid	0.5067290818	0.3107830593	0.3092153268	0.2112351178
Ω-muricholic acid	0.5583040831	0.3510414675	0.3786051215	0.2790380631

Supplemental Table 3

**Q-values**	CD11c (SAT)	F4/80 (SAT)	insulin (pM)	HOMA-IR
Isolithocholic acid	0.8539111154	0.9425492642	0.4710280534	0.4551084466
Taurochenodeoxycholic acid	0.072438496	0.0574390035	0.0003225753	0.0002563707
Murocholic acid	0.9663363187	0.8133718215	0.5070564464	0.4710280534
Taurolithocholic acid	0.6932538849	0.6093415927	0.7048720495	0.6093415927
Chenodeoxycholic acid	0.2748472191	0.3621486305	0.5070564464	0.493783153
12-Ketochenodeoxycholic acid	0.6984929375	0.548502293	0.548502293	0.493783153
Hyodeoxycholic acid	0.9486253467	0.8764376004	0.4710280534	0.4710280534
Taurohyocholic acid	0.4551084466	0.4551084466	0.9411848979	0.8133718215
Hyocholic acid	0.7650803377	0.5703232047	0.493783153	0.4710280534
Lithocholic acid	0.8133718215	0.6876181725	0.493783153	0.4710280534
12-Ketolithocholic acid	0.7845667269	0.9425492642	0.4551084466	0.4027675836
7-Ketodeoxycholic acid	0.8214039858	0.9751875061	0.4551084466	0.4551084466
Ursodeoxycholic acid	0.6984929375	0.6152458293	0.5070564464	0.4710280534
Taurohyodeoxycholic and Tauroursodeoxycholic acid	0.2489577159	0.265111887	0.4809680203	0.4813086
Taurodeoxycholic acid	0.548502293	0.4710280534	0.5070564464	0.4839960822
Allocholic acid	0.493783153	0.4643866699	0.4710280534	0.4643866699
Taurocholic acid	0.0684476798	0.0574390035	0.0003225753	0.0002563707
β-muricholic acid	0.5899177757	0.548502293	0.6876181725	0.5600909221
Tauro-α-muricholic acid	0.2102721837	0.2970336984	0.4551084466	0.4643866699
Cholic acid	0.4710280534	0.493783153	0.8920986514	0.7449117895
Tauro-ω-muricholic acid	0.4710280534	0.4839960822	0.8133718215	0.7588595162
α-muricholic acid	0.4710280534	0.4643866699	0.8133718215	0.6876181725
Tauro-β-muricholic acid	0.548502293	0.5704098133	0.1156255177	0.1156255177
Deoxycholic acid	0.6876181725	0.5358328608	0.5358328608	0.4839960822
Ω-muricholic acid	0.6984929375	0.548502293	0.5703232047	0.5070564464

Supplemental Table 4

	LFD D12450K		WD modified D12451		RS2		RS4	
	**gm%**	**kcal%**	**gm%**	**kcal%**	**gm%**	**kcal%**	**gm%**	**kcal%**
**Protein**	**19.2**	**20.0**	**23.7**	**20.0**	**23.7**	**21.9**	**23.7**	**21.9**
**Carbohydrate**	**67.3**	**70.0**	**46.1**	**34.1**	**46.1**	**28.0**	**46.1**	**28.0**
**Fat**	**4.3**	**10.0**	**23.6**	**44.9**	**23.6**	**49.1**	**23.6**	**49.1**
Total		100.0	93.4	99.0	93.4	98.9	93.4	98.9
kcal/gm	3.8		4.7		4.3		4.3	
**Ingredients**	**gm**	**kcal**						
Casein, 30 Mesh	200.0	800.0	200.0	800.0	200.0	800.0	200.0	800.0
L-cystine	3.0	12.0	3.0	12.0	3.0	12.0	3.0	12.0
Corn Starch	550.0	2200.0	137.3	549.2	0.0	0.0	41.0	164.0
Maltodextrine 10	150.0	600.0	35.5	142.0	35.5	142.0	35.5	142.0
Sucrose	0.0	0.0	172.8	691.0	172.8	691.0	172.8	691.0
HiMaize (RS2)	0.0	0.0	0.0	0.0	137.3	206.0	0.0	0.0
Fybersym (RS4)	0.0	0.0	0.0	0.0	0.0	0.0	96.3	42.0
Cellulose microcristalline	0.0	0.0	0.0	0.0	0.0	0.0	0.0	0.0
Cellulose BW200	50.0	0.0	50.0	0.0	50.0	0.0	50.0	0.0
Soybean Oil	25.0	225.0	25.0	225.0	25.0	225.0	25.0	225.0
Lard	20.0	180.0	177.5	1598.0	177.5	1598.0	177.5	1598.0
Mineral Mix S10026	10.0	0.0	10.0	0.0	10.0	0.0	10.0	0.0
DiCalcium Phosphate	13.0	0.0	13.0	0.0	13.0	0.0	13.0	0.0
Calcium Carbonate	5.5	0.0	5.5	0.0	5.5	0.0	5.5	0.0
Potassium Citrate, 1H2O	16.5	0.0	16.5	0.0	16.5	0.0	16.5	0.0
Vitamin Mix V1001	10.0	40.0	10.0	40.0	10.0	40.0	10.0	40.0
Choline Bitartrate	2.0	0.0	2.0	0.0	2.0	0.0	2.0	0.0
Dyes	0.1	0.0	0.1	0.0	0.1	0.0	0.1	0.0
Total	1055.1	4057.0	858.2	4057.2	858.2	3714.0	858.2	3714.0
