# Supplementary material for: Resistant starch can improve insulin sensitivity independently of the gut microbiota
Source: Microbiome. 2017 Feb 7;5:12. doi: 10.1186/s40168-017-0230-5 (PMC5294823; doi:10.1186/s40168-017-0230-5)
Supplement: Additional file 6: — Supplemental materials and methods. (PDF 208 kb) [file 40168_2017_230_MOESM6_ESM.pdf]

Resistant starch can improve insulin sensitivity independently of the gut microbiota

SUPPLEMENTAL MATERIALS AND METHODS

Mice. For conventionalization, cecal contents were collected aseptically from 8 to 15 conventionally-raised (CONV) C3H/HeN donors bred at UNL, pooled into sterile phosphate buffered saline, and then transferred to GF C3H (5.5 wk old) and C57BL/6 (B6; 8 wk old) male mice via oral gavage. Studies were designed such that all mice, regardless of their genetic background, possessed a similar body weight at baseline, thus explaining the age difference between the two mouse strains. CONV B6 mice were also orally gavaged with a cecal slurry from CONV C3H mice 9 d prior to experimental diet introduction. CONV and CVZ mice were subsequently housed in autoclaved, individually ventilated cages. Germ-free (GF) littermates were housed in flexible-film isolators and maintained under gnotobiotic conditions for the duration of the experiments.

Mice were fasted for 6 h prior to receiving an intraperitoneal injection of insulin (1 U/kg BW, Novolin R, Novo Nordisk, Princeton, NJ) for an insulin tolerance test performed at week 7. Blood glucose levels were determined using a glucose meter (Roche Diagnostics, Indianapolis, IN) and blood collected from the tip of the tail at the indicated time points. Blood was also sampled from mice fasted for 6 hours prior to necropsy to measure glucose and insulin levels.

Gut barrier integrity was assessed by administering 4 kDa FITC-dextran (Sigma, St. Louis, MO) via oral gavage (600 mg/kg BW; 125 mg/mL of phosphate buffered saline) to 6-hour fasted mice one hour before necropsy (1). Plasma collected at necropsy was diluted 1:1 with PBS and fluorescence was measured using a spectrofluorometer (485 nm excitation and 535 nm emission). Standard curves were generated by serially diluting FITC-dextran in non-treated plasma with PBS.

Tissue analyses. Adipose tissues, livers, ileum, colon and cecal tissues and contents were frozen in liquid nitrogen. Visceral adipose tissue was dissected as described by Caesar *et al.*(2). A portion of subcutaneous and visceral adipose tissue was fixed in RNA Later (Ambion, Life Technologies, Grand Island, NY). All samples were stored at -80°C until further use. Gene expression analyses were performed as previously described (3) using RealMasterMix SYBR ROX (5Prime, Gaithersburg, MD) or Maxima SYBR Green qPCR Master Mix (Thermo Scientific, Waltham, MA) and a Mastercycler ep realplex (Eppendorf AG, Hamburg, Germany) for detection and RPL4 as a housekeeping gene. RNA quality was checked for a subset of samples using an Agilent 2100 Bioanalyzer according to manufacturer's instructions (Agilent Technologies, Santa Clara, CA). For visceral adipose tissue, all samples were analyzed for RNA quality. Eight out of the 63 samples of visceral adipose tissue presented a RIN <5 and were discarded from the analyses. Primer sequences are provided in Table A.

Flow cytometry. Flow cytometric analysis of the stromal vascular fraction (SVF) from subcutaneous adipose tissue was performed using published protocols (4-6). Briefly, lymph nodes were removed and one g of adipose tissue was minced in KREB-BSA, digested using Collagenase I (Worthington, Lakewood, NJ) for 40 min at 37°C, filtered and washed. Red blood cells were removed using ACK lysis buffer. SVF cells were quantified using a cellometer with acridine orange and propidium iodide (Nexcelom Bioscience LLC, Lawrence, MA) prior to being labeled with PE F4/80 (clone BM8), PE-Cy7 CD11c (clone N418), APC-Cy7 CD11b (clone M1/70), PerCP-Cy5.5 CD206 (clone C068C2; Biolegend, San Diego, CA), APC CD301 (clone ER-MP23; AbD Serotec, Raleigh, NC) and PE-Texas Red CD45.2 (clone 104). All antibodies were obtained from eBioscience (San Diego, CA) unless otherwise noted. Cells were analyzed using a BD FACSAria

III (BD Biosciences, San Jose, CA). Data were analyzed using FlowJo X 10.0.7 (Tree star, Ashland, OR); gates were drawn based on fluorescence minus one controls.

Microbial community analysis. Gut microbiota composition was assessed by 16S rRNA gene sequencing of fecal samples as previously described (7). Genomic DNA was extracted from feces using a QIAamp DNA Stool Mini Kit (Qiagen, Valencia, CA) with a bead-beating step.

Amplicon sequencing of the fecal microbiota was done at the University of Minnesota Genomics Center, as described in the supplemental methods. Briefly, the V5-V6 region of the 16S rRNA gene was PCR-enriched using the primer pair 784F (5'-RGGATTAGATACCC-3') and 1064R (5'-CGACRRCCATGCANCACCT-3') in a 25 µL PCR reaction containing 5 µL of template DNA, 5 µL of 2X HotStarTaq *Plus* Master Mix, 500 nM of final concentration of primers and 0.025 U/µL of HotStarTaq *Plus* DNA Polymerase (QIAGEN). PCR-enrichment reactions were conducted as follows, an initial denaturation step at 95°C for 5 min followed by 20-25 cycles of denaturation (50 s at 94°C), annealing (30 s at 40°C), and elongation (30 s at 72°C).

Next, the PCR-enriched samples were diluted 1:100 in water for input into library tailing PCR. The PCR reaction was analogous to the one conducted for enrichment except for a Taq polymerase concentration of 0.25 U/µL. The cycling conditions used were as follows: initial denaturation at 95°C for 5 min followed by 10-15 cycles of denaturation (50 s at 94°C), annealing (30 s at 40°C), and elongation (1 min at 72°C). The primers used for tailing were the following: F-indexing primer AATGATACGGCGACCACCGAGATC TACAC[i5]TCGTCGGCAGCGTC and R-indexing primer CAAGCAGAAGACGGCATACG AGAT[i7]GTCTCGTGGGCTCGG, where [i5] and [i7] refer to the index sequence codes used by Illumina.

The resulting PCR products were quantified by PicoGreen (Life Technologies). A subset of the amplicon libraries was spot-checked on a Bioanalyzer High-Sensitivity DNA Chip (Agilent

Technologoies) for correct amplicon size. Next, samples were normalized to 2nM and pooled together. The total volume of the libraries was reduced by SpeedVac and amplicons were size-selected at 420 bp +/- 20% using the Caliper XT (Perkin Elmer). Next, library pools were cleaned-up by 1.8X AMPureXP beads (Beckman Coulter) and eluted in water. The final pool was quantified by PicoGreen and normalized to 2 nM for input into Illumina MiSeq (v3 Kit) to produce 2x300 bp sequencing products. Clustering was done at 10 pM with a 5% spike of PhiX.

The sequences used for analysis can be found in the MG-RAST database (8) with the following accession numbers: 4603180.3, 4603181.3, 4603182.3, 4603183.3, 4603184.3, 4603185.3, 4603186.3, 4603187.3, 4603188.3, 4603189.3, 4603190.3, 4603191.3, 4603192.3, 4603193.3, 4603194.3, 4603195.3, 4618881.3, 4618882.3, 4618883.3, 4618884.3, 4618885.3, 4618886.3, 4618887.3, 4618888.3, 4618889.3, 4618890.3, 4618891.3, 4618892.3, 4618893.3, 4618894.3, 4618895.3, 4618896.3.

Bile acids analysis. Forty-six bile acids were quantified by ultrahigh performance liquid chromatography – multiple-reaction monitoring mass spectrometry (UPLC-MRM-MS) at the University of Victoria Genome British Columbia Proteomics Centre using a protocol adapted from Han et al. (9). In brief, the cecal contents were homogenized in water at a ratio of 150 µL per 10 mg raw material at a shaking frequency of 30 Hz for 1 min twice with the aid of two 5-mm stainless steel metal balls using a Restch MM 400 mixer mill (Haan, Germany). Bile acids were extracted by addition of acetonitrile at a ratio of 350 µL per 10 mg raw material followed by vortex mixing and 1-min sonication in an ice water bath. The samples were centrifuged at 12,500 rpm for 10 min at 4°C. Twenty µL of each supernatant were mixed with 50 µL of an internal standard (IS) solution that contained 0.05 to 0.2 nmol/mL of 14 deuterium (D)-labeled bile acids (Table B). After addition of 430 µL of 70% acetonitrile and 10 µL of 10% formic acid, the mixtures were cleaned up by

phospholipid-depletion solid-phase extraction on a HybridSPE®-Phospholipid 96-well plate (50 mg/2 mL; Sigma-Aldrich) using the same procedure as described in (13). The flow-through fractions were collected and then dried under a gentle nitrogen flow. The residues were dissolved in 200 μL of 50% methanol. Ten-μL aliquots were injected. For the analysis of the high abundance bile acids, the samples were diluted 25 times and then re-injected. The UPLC-MRM/MS analyses were performed using a Dionex UltiMate 3400 RSLC system (Amsterdam, The Netherlands) coupled to an AB Sciex 4000 QTRAP mass spectrometer (Concord, ON, Canada) equipped with a TurboIon electrospray ionization (ESI) source and operated in the negative ion multiple-reaction monitoring (MRM) mode. UPLC separation was carried out on a Waters BEH C_{18} UPLC column (2.1 x 150 mm, 1.7 μm) with water-acetonitrile-0.01% formic acid as the mobile phase for binary gradient elution using the same UPLC and MRM/MS parameters and operating procedures as described in (9).

Concentrations of the detected bile acids (Table B) were calculated with internal calibration from their linearly regressed standard curves prepared using the authentic compounds of 46 bile acids. Since taurohyodeoxycholic acid and tauroursodeoxycholic acid were not resolved by UPLC-MRM/MS, the total amount of these two compounds in each sample was reported as the concentration of tauroursodeoxycholic acid. The 14 D-labeled bile acids were used as IS for their corresponding non-D-labeled forms. For the bile acids without their D-labeled analogues, chenodeoxycholic-D_4 acid was used as the common IS for quantitation of the unconjugated species; tauro-chenodeoxycholic-D_4 acid was used as the common IS for quantitation of the taurine-conjugated species; glyco-deoxycholic-D_4 acid was used as the common IS for quantitation of the glycine-conjugated species. Since there were no standard compounds available for glyco-ω-muricholic acid (MCA), glyco-α-MCA, glyco-β-MCA or glyco-allocholic acid, the identities of these compounds, which shared the same MRM transitions as those of their isomeric glycocholic

acid, were deduced from the recorded chromatographic retention times by comparison of the retention times of their corresponding unconjugated and taurine-conjugated species on the acquired UPLC-MRM/MS profiles. The concentrations of glyco-ω-MCA, glyco-α-MCA, glyco-β-MCA, and glyco-allocholic acid were calculated from the calibration curve of glycocholic acid. In the analysis, the lower limits of quantification were 0.005 nmole/g.

Table A. Primer sequences used for q-PCR.

	**Forward primer**	**Reverse primer**
RPL4 (housekeeping gene)	CAAGAAGACCAAGGAGGCTGT	GGTTTCTCATTTTGCCCTTG
F4/80	TGACAACCAGACGGCTTGTG	CAGGCGAGGAAAAGATAGTGT
CD11c	ACGTCAGTACAAGGAGATGTTGGA	ATCCTATTGCAGAATGCTTCTTTACC
Proglucagon	TGGCAGCACGCCCTTC	GCGCTTCTGTCTGGGA
PYY	GTTTGGACCAGTGGTGAAGA	TGCCCTCTTCTTAAACCAAACA
PGC1α	AGCCGTGACCACTGACAACGAG	AGCCGTGACCACTGACAACGAG
CPT1α	AGACCGTGAGGAACTCAAACCTAT	TGAAGAGTCGCTCCCACT
PCK1	AGAACAAGGAGTGGAGACCG	AGGGCTTCATAGACAAGGGG
FAS	AGATGAAGGTGGCAGAGGTG	GTAGCATGGGCTGGGTGTT
ACC	TGTTGAGACGCTGGTTTGTAGAA	GGTCCTTATTATTGTCCCAGACGTA
G6Pase	CTGTCTGTCCCGGATCTACC	GCGCGAAACCAAACAAGAAG
ZO-1	TTTTTGACAGGGGGAGTGG	TGCTGCAGAGGTCAAAGTTCAAG
Occludin	AGGCAGAACTAGACGACGTC	TGCTCTTCAACTGCTTGCAG
adipoQ	GGAGATGCAGGTCTTCTTGG	GCTTCTCCAGGCTCTCCTTT
MCP-1	GCAGTTAACGCCCCACTCA	CCCAGCCTACTCATTGGGATCA

Table B. Analyzed bile acids with their abbreviations

12-KCDCA	12-Ketochenodeoxycholic acid
12-KLCA	12-Ketolithocholic acid
23-NDCA	23-Nordeoxycholic acid
3-OCA	3-Oxocholic acid
6,7-DiKLCA	6,7-Diketolithocholic acid
7-KDCA	7-Ketodeoxycholic acid
7-KLCA	7-Ketolithocholic acid
ACA	Allocholic acid
AILCA	Alloisolithocholic acid
aMCA	α-muricholic acid
ApoCA	Apocholic acid
bMCA	β-muricholic acid
CDCA	Chenodeoxycholic acid*
CA	Cholic acid*
DCA	Deoxycholic acid*
DHCA	Dehydrocholic acid
DHLCA	Dehydrolithocholic acid
DioLCA	Dioxolithocholic acid
GalloCA	Glyco-allocholic acid
GaMCA	Glyco-α-muricholic acid
GbMCA	Glyco-β-muricholic acid
GCA	Glycocholic acid*
GCDCA	Glycochenodeoxycholic acid*
GDCA	Glycodeoxycholic acid*
GHCA	Glycohyocholic acid
GLCA	Glycolithocholic acid*
GUDCA	Glycoursodeoxycholic acid*
GwMCA	Glyco-ω-muricholic acid
HCA	Hyocholic acid
HDCA	Hyodeoxycholic acid
IDCA	Isodoxycholic acid
ILCA	Isolithocholic acid
LCA	Lithocholic acid*
MuroCA	Murocholic acid
TaMCA	Tauro-α-muricholic acid
TbMCA	Tauro-β-muricholic acid
TCA	Taurocholic acid*
TCDCA	Taurochenodeoxycholic acid*
TDCA	Taurodeoxycholic acid*

TDHCA	Taurodehydrocholic acid
THCA	Taurohyocholic acid
THDCA+TUDCA	Taurohyodeoxycholic and Tauroursodeoxycholic acid*
TLCA	Taurolithocholic acid
TwMCA	Tauro-ω-muricholic acid
UCA	Ursocholic acid
UDCA	Ursodeoxycholic acid*
wMCA	ω-muricholic acid

*Deuterium-labeled bile acids used as internal standards for quantification (all D4, except for Taurodeoxycholic-D6 acid).

SUPPLEMENTAL REFERENCES

1. **Cani PD, Possemiers S, Van de Wiele T, Guiot Y, Everard A, Rottier O, Geurts L, Naslain D, Neyrinck A, Lambert DM, Muccioli GG, Delzenne NM.** 2009. Changes in gut microbiota control inflammation in obese mice through a mechanism involving GLP-2-driven improvement of gut permeability. Gut **58**:1091-1103.

2. **Caesar R, Drevon CA.** 2008. Pancreatic contamination of mesenteric adipose tissue samples can be avoided by adjusted dissection procedures. J Lipid Res **49**:1588-1594.

3. **Bindels LB, Beck R, Schakman O, Martin JC, De Backer FC, Sohet FM, Dewulf EM, Pachikian BD, Neyrinck AM, Thissen JP, Verrax J, Calderon PB, Pot B, Grangette C, Cani PD, Delzenne NM.** 2012. Restoring Specific Lactobacilli Levels Decreases Inflammation and Muscle Atrophy Markers in an Acute Leukemia Mouse Model. PLoS One **7**:e37971.

4. **Cho KW, Morris DL, Lumeng CN.** 2014. Flow cytometry analyses of adipose tissue macrophages. Methods Enzymol **537**:297-314.

5. **Brake DK, Smith CW.** 2008. Flow cytometry on the stromal-vascular fraction of white adipose tissue. Methods Mol Biol **456**:221-229.

6. **Romeo GR, Pae M, Eberle D, Lee J, Shoelson SE.** 2013. Profilin-1 haploinsufficiency protects against obesity-associated glucose intolerance and preserves adipose tissue immune homeostasis. Diabetes **62**:3718-3726.

7. **Krumbeck JA, Maldonado-Gomez MX, Martinez I, Frese SA, Burkey TE, Rasineni K, Ramer-Tait AE, Harris EN, Hutkins RW, Walter J.** 2015. Introducing the concept of In Vivo Selection to identify bacterial stains with enhanced ecological performance when used in synbiotic applications. Appl Environ Microbiol **81**:2455-2465.

8. **Meyer F, Paarmann D, D'Souza M, Olson R, Glass EM, Kubal M, Paczian T, Rodriguez A, Stevens R, Wilke A, Wilkening J, Edwards RA.** 2008. The metagenomics RAST server - a public resource for the automatic phylogenetic and functional analysis of metagenomes. BMC Bioinformatics **9**:386.

9. **Han J, Liu Y, Wang R, Yang J, Ling V, Borchers CH.** 2015. Metabolic Profiling of Bile Acids in Human and Mouse Blood by LC-MS/MS in Combination with Phospholipid-Depletion Solid-Phase Extraction. Anal Chem **87**:1127-1136.
